# Advanced glycation end products promote meniscal calcification by activating the mTOR-ATF4 positive feedback loop

**DOI:** 10.1038/s12276-024-01190-6

**Published:** 2024-03-01

**Authors:** Sheng Yang, JiaJun Xie, ZhiJie Pan, HongMei Guan, YueSheng Tu, YuanJian Ye, ShouBin Huang, ShiQiang Fu, KangXian Li, ZhiWei Huang, XiaoQi Li, ZhanJun Shi, Le Li, Yang Zhang

**Affiliations:** 1 Division of Orthopaedic Surgery, Department of Orthopaedics, Nanfang Hospital, Southern Medical University, Guangzhou, Guangdong China; 2 Department of Orthopedics, 920 Hospital of the Joint Logistic Support Force, Kunming, Yunnan China; 3 Department of Orthopaedics, The First People’s Hospital of Zhaoqing, Zhaoqing, Guangdong China; 4 Department of Obstetrics and Gynecology, Guangdong Provincial Key Laboratory of Major Obstetric Diseases, The Third Affiliated Hospital of Guangzhou Medical University, Guangzhou, China; 5 Department of Orthopaedics, Huizhou First Hospital, Guangdong Medical University, Huizhou, Guangdong China; 6 Huizhou First Maternal and Child Health Care Hospital, Huizhou, Guangdong China; 7 The First School of Clinical Medicine, Southern Medical University, Guangzhou, Guangdong China; 8 School of Public Health, Southern Medical University, Guangzhou, Guangdong China; 9 Department of Anesthesiology, Zhujiang Hospital, Southern Medical University, Guangzhou, Guangdong China

**Keywords:** Post-translational modifications, Cell signalling

## Abstract

The meniscus is vital for maintaining knee homeostasis and function. Meniscal calcification is one of the earliest radiological indicators of knee osteoarthritis (KOA), and meniscal calcification is associated with alterations in biomechanical properties. Meniscal calcification originates from a biochemical process similar to vascular calcification. Advanced glycation end products (AGEs) and their receptors (RAGEs) reportedly play critical roles in vascular calcification. Herein, we investigated whether targeting AGE-RAGE is a potential treatment for meniscal calcification. In our study, we demonstrated that AGE-RAGE promotes the osteogenesis of meniscal cells and exacerbates meniscal calcification. Mechanistically, AGE-RAGE activates mTOR and simultaneously promotes ATF4 accumulation, thereby facilitating the ATF4-mTOR positive feedback loop that enhances the osteogenic capacity of meniscal cells. In this regard, mTOR inhibits ATF4 degradation by reducing its ubiquitination, while ATF4 activates mTOR by increasing arginine uptake. Our findings substantiate the unique role of AGE-RAGE in the meniscus and reveal the role of the ATF4-mTOR positive feedback loop during the osteogenesis of meniscal cells; these results provide potential therapeutic targets for KOA.

## Unlocking Knee Osteoarthritis: AGEs-RAGE’s Role in Meniscal Calcification

In our joints, the meniscus is vital for knee stability and function. Sadly, when the meniscus hardens, it can result in a painful ailment called knee osteoarthritis. This study, led by researchers including Yang Zhang, sought to comprehend the early stages of this hardening process to find treatments before it inflicts substantial damage. This research was an experiment involving human tissue samples and cell cultures to understand the disease mechanisms at a cellular level. The findings indicated that higher AGEs levels correlated with more severe hardening. Researchers hope that their work will lead to new treatments that can prevent or slow down the progression of this debilitating condition. Future studies could build on these findings to develop therapies that help maintain knee health and mobility.

This summary was initially drafted using artificial intelligence, then revised and fact-checked by the author.

## Introduction

The meniscus plays an essential role, biochemically and biomechanically, in maintaining the homeostasis and normal function of the knee^1,2^. Current evidence suggests that calcification of the meniscus is one of the major causes of knee osteoarthritis (KOA) since it alters the biomechanical properties of the knee, resulting in upregulated expression of inflammatory genes, cytokines and matrix metalloproteinases (MMPs)^3^. For symptomatic OA, the estimated lifetime risk of KOA is ~14%^4^. In the USA, the total annual average direct costs varied from US$1442 to US$21,335, resulting in a heavy socioeconomic burden^5^.

Currently, the primary goal of KOA treatment is pain relief^6^. Few effective treatments are available to help prevent the progression of KOA, given that the structure of the knee is typically significantly damaged when a clinical diagnosis is made. Most patients with KOA tend to progress to an advanced stage where pain and stiffness become intolerable and patients require surgery. However, the efficacy of surgery is limited, and surgery is accompanied by serious complications or unsatisfactory functional recovery^7^. Thus, early identification and therapeutic intervention represent major challenges in the treatment of KOA.

Various studies have shown that meniscal calcification may be the earliest radiological sign of KOA, and that meniscal calcification further leads to cartilage damage and accelerates the progression of KOA^8–10^. Therefore, targeting meniscal calcification is a promising treatment option in the early stage of KOA. Several studies have been performed to identify the causes of meniscal calcification. Ho et al. reported that deletion of the calcification plasma cell membrane glycoprotein-1 (PC-1) resulted in extensive meniscal calcification^11^. In humans, proton pump inhibitors can inhibit calcium deposition in OA meniscal cells^12^. Overwhelming evidence substantiates the close relationship between cellular hypertrophy and calcification in the meniscus^13^. However, to date, the pathogenesis of meniscal calcification has not been determined.

Recent evidence suggests that meniscal calcification may originate from a biochemical process mediated by a combination of systemic and local factors^8^, similar to the pathological process of vascular calcification. The active osteogenic process of vascular cells, resembling osteoblast formation, is the leading cause of vascular calcification^14,15^. Studies have shown that advanced glycation end products (AGEs) and their receptors (RAGEs) play important roles in the osteogenesis of vascular cells^16,17^. An increasing body of evidence suggests that the AGE-RAGE pathway may initiate osteogenesis by regulating the transdifferentiation of vascular smooth muscle cells to osteoblast-like cells^18,19^. Furthermore, AGEs induce irreversible crosslinking between matrix collagen and elastin, which results in a stiffer extracellular matrix^20^. Interestingly, local accumulation of AGEs has been documented early in the process of knee degeneration^21–23^, which results in cartilage destruction^24,25^. Thus, we speculate that AGEs play a critical role in regulating meniscal calcification.

In this study, we investigated the potential of the AGE-RAGE signaling pathway to promote osteogenesis and cause meniscal calcification and investigated the underlying mechanisms involved. We identified a novel positive feedback loop between mTOR and ATF4. To the best of our knowledge, this is the first study to investigate the mechanism underlying meniscal calcification and the underlying processes. These findings shed new light on a potential therapeutic strategy for early KOA.

## Materials and methods

### Human meniscus samples

This study was approved by the Ethics Committees of Nanfang Hospital, Southern Medical University (NFEC-2022-499). Human meniscus samples were obtained from 20 patients with knee OA who underwent knee arthroplasty surgery. We evaluated the severity of meniscal degeneration using Paudi’s method^26^. The histological assessment indicated that the meniscus tissues from OA patients exhibited Grade 3 (moderate degeneration) or Grade 4 (severe degeneration) severity. All the participants were divided into two groups according to their HbA1c level (low HbA1c group (*n* = 10) vs. high HbA1c group (*n* = 10), 5.81 ± 0.246 vs. 7.04 ± 1.01, *P* < 0.001). These two groups of patients were similar in age (65.7 ± 6.67 vs. 67.3 ± 4.56 years, *P* = 0.55). Moreover, the chi-square test showed no significant difference in the sex ratio between the two groups (*P* = 0.19). We were able to collect two meniscus samples from each patient. The medial and lateral meniscal tissues were cut into small coronal sections and preserved separately. We used the anterior horn of the meniscus in this study because meniscal tissues were harvested during TKA procedures, during which a medial patellar approach was applied. Consequently, the anterior horn was the most integral part of the sample, which was helpful for quality control of the experiments. For the samples that were subjected to μCT scanning and sectioning (from the lateral meniscus), we fixed them in 4% paraformaldehyde for 24 h immediately after collection and then washed and stored them in 70% ethanol to preserve antigen information. After performing μCT scans, the samples were subjected to subsequent sectioning and staining. The other samples (medial meniscus) were preserved in RNAlater (Thermo, AM7021) for protein and RNA extraction.

### Isolation and culture of human meniscal cells

The meniscus samples used to extract primary meniscal cells were obtained from 20 male patients (aged 69.8 ± 4.99 years) with knee OA who underwent knee arthroplasty surgery. The histological assessment indicated that the meniscus tissues from OA patients exhibited Grade 3 (moderate degeneration) or Grade 4 (severe degeneration) severity according to Paudi’s method^26^. The HbA1c level was normal in these patients (5.46 ± 0.29).

Human meniscus tissues were dissected away from the synovium and cut into small pieces under sterile conditions. Next, these small pieces were digested with 4 mg/mL protease (11459643001; Roche) for 1 h and 2 mg/mL collagenase P (11213873001; Roche) for 6–10 h. Meniscal cells were obtained after sterile filtering and were cultured in DMEM/Nutrient Mixture F-12 (Gibco) supplemented with 5% fetal bovine serum (ExCell), 100 IU/mL penicillin, and 100 μg/mL streptomycin (Beyotime). First- to third-generation cells were used for this research to avoid phenotypic loss.

For osteogenic differentiation, the media was changed to osteogenic media (plating media supplemented with 50 μM ascorbic acid, 10 mM phosphate and 100 nM dexamethasone). After 7 days, the cells were stained with ALP (for visualization of osteoblasts) in differentiation media or with Alizarin Red (for visualization of mineral formation) after 21 days.

### Animals

The animal protocol was approved by the Animal Care Committee of Southern Medical University. All the experimental protocols used were approved by The State Council of the People’s Republic of China “Experimental Animal Administration Regulations”. Male C57BL/6J (RRID: IMSR_JAX:000664) mice were purchased from the Animal Center of Southern Medical University. The BKS. Cg-Dock7^m^ +/+ Lepr^db^/J mice (RRID: IMSR_JAX:000642) were obtained from Jackson Laboratory and are among the most widely used diabetes models. In brief, mice homozygous for the spontaneous diabetes mutation (db/db for short) became identifiably obese at approximately three to 4 weeks of age. Elevations in plasma insulin began at 10–14 days of age, and fluctuations in blood sugar began at four to eight weeks. Mice that were heterozygous (db/m for short) had a normal body weight, blood glucose concentration, and plasma insulin concentration.

### Chemicals and reagents

The RAGE inhibitor TTP488 (HY-50682, MedChemExpress), the PI3K inhibitor Copanlisib (BAY 80-6946, MedChemExpress), the AKT inhibitor MK2206 (HY-108232, MedChemExpress), the mTOR inhibitor rapamycin (HY-10219, MedChemExpress), the mTOR agonist MHY1485 (HY-B0795, MedChemExpress) and the proteasome agent MG132 (HY-13259, MedChemExpress) were dissolved in DMSO as stock solutions and stored at −80 °C. The proteins were diluted to a working solution in culture medium before use. AGEs (bs-1158P, Bioss) were not dissolved in DMSO, and the stock solution was provided in liquid form. We diluted the solution to a working solution using culture medium before use. The working solution concentrations were determined by preliminary experiments, and the solution did not cause significant cell death (Supplementary Fig. 9). When the reagents were processed into working solutions, we added different amounts of DMSO depending on the concentration of the stock solution so that the concentration of DMSO in the working solution was 1/1000 (v/v). The specific concentrations are provided in Supplementary Table 1.

### Anterior cruciate ligament transection (ACLT)-induced OA model

Mice underwent the indicated surgical procedure (sham or ACLT) at 10 weeks using a small animal anesthesia machine and a surgical table in the SPF animal room. For ACLT surgery, after opening the joint capsule, the ACL was transected with microscissors under a surgical microscope. After the operation was complete, the animals were allowed to rest for 1 week, after which the indicated drugs (working solution) or culture media (control) were injected intra-articularly into the knee. The injections were performed using a microsyringe with 10 µl of drug per knee joint. Intra-articular knee injections were performed every 4 days. The knee joint was harvested after 8 weeks of injection.

### Microcomputed tomography (μCT)

Specimens were scanned using a Bruker Micro-CT Skyscan 1276 system (Kontich) using the following settings: voxel size, 6.5 μm; medium resolution, 85 kV; 200 mA; 1 mm Al filter; and integration time, 384 ms. Density measurements were calibrated to the manufacturer’s calcium hydroxyapatite (CaHA) phantom. Analysis was performed using the manufacturer’s evaluation software. Reconstruction was accomplished by NRecon (version 1.7.4.2). 3D images were obtained from contoured 2D images based on the distance transformation of the original grayscale images (CTvox; version 3.3.0).

**Fig. 1:**
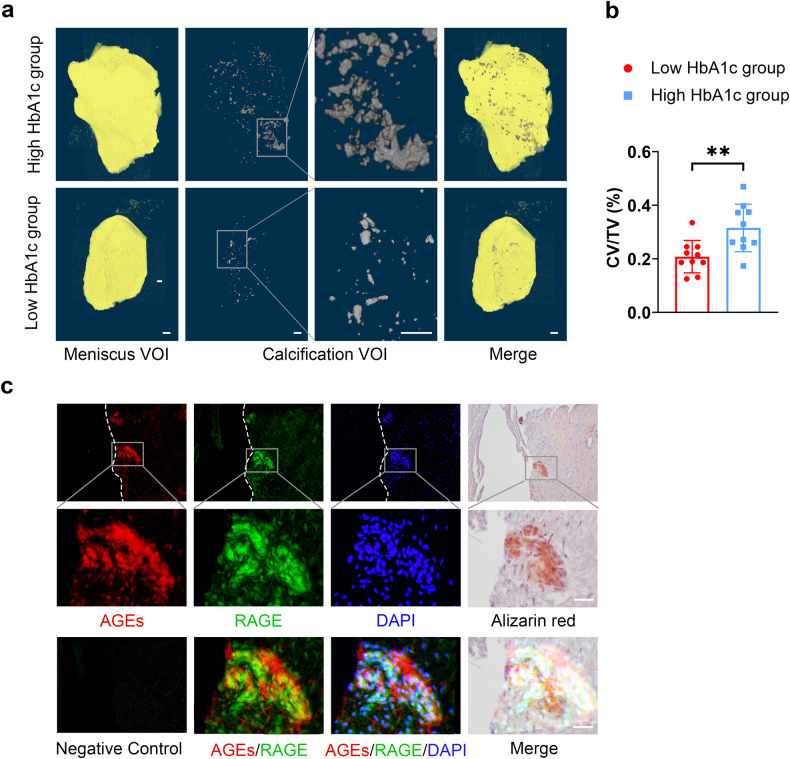
AGEs are positively correlated with meniscal calcification. **a** Micro-CT reconstruction of meniscus VOI (volume of interest) and calcified VOI. Scale bar: 1.25 mm. **b** Quantification of the total tissue volume (TV) and calcification volume (CV) of the meniscus was performed using CTAn software; *n* = 10. **c** Alizarin red staining and AGE and RAGE immunofluorescence (IF) staining of meniscus sections. Scale bar: 25 μm. The data are expressed as the mean ± SD; *P < 0.05; **P < 0.01; and ***P < 0.001. Student’s *t* test was used for comparison.

**Fig. 2:**
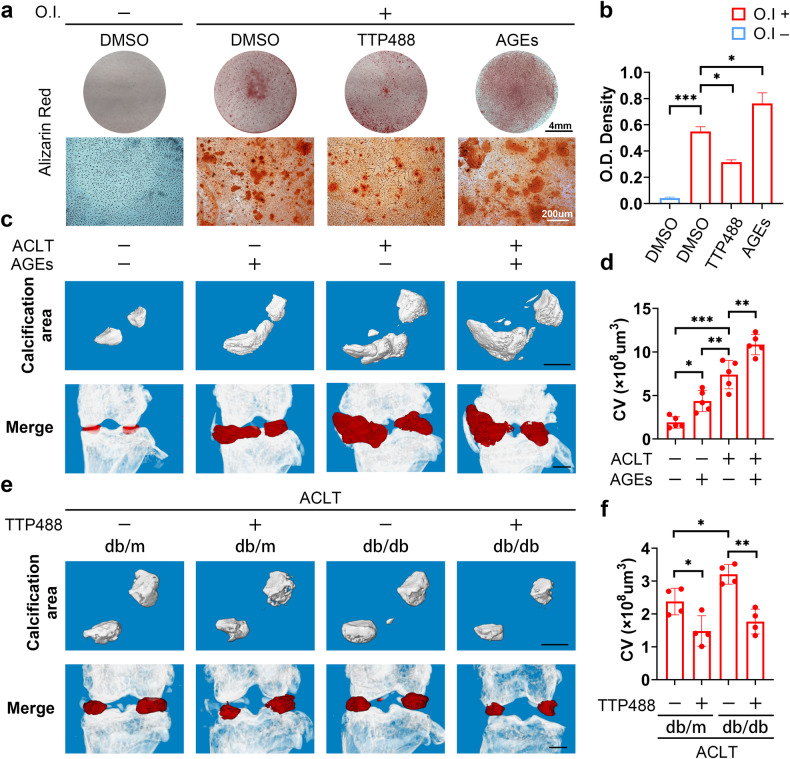
Activation of AGE-RAGE signaling enhances the osteogenic differentiation and mineralization of meniscal cells. **a** Human primary meniscal cells were treated with the indicated drugs during osteogenic induction, and Alizarin Red was used to detect osteogenic differentiation. **b** Quantification of Alizarin Red staining; *n* = 3. **c**, **e** Three-dimensional micro-CT images of the calcification area in the meniscal region and merged images with the knee joints in different mouse groups. Scale bar: 1 mm. **d** Quantification of the calcification volume; *n* = 5. **f** Quantification of the calcification volume; *n* = 4. O.I., osteogenic induction (21 days). The data are expressed as the mean ± SD; *P < 0.05; **P < 0.01; and ***P < 0.001. One-way ANOVA was used for comparisons.

For the analysis of the calcification volume of clinical meniscal tissue samples (Fig. 1), we used CT Analyzer (version 1.18.8.0) to select the range where meniscal tissue appeared as ROIs (regions of interest) in the “regions of interest preview”. Subsequently, we set the lower gray threshold to 90 and the upper gray threshold to 255 using the “binary selection preview” function to select the calcification range. Finally, the calcification volume percentage (CV/TV) was calculated using the “3D analysis” function in “Custom processing”. For the analysis of the meniscal calcification volume (Fig. 2) and subchondral bone BV/TV (Supplementary Fig. 2) of the mice, we adopted the same method, but when selecting the ROI, we chose the area where the meniscus and subchondral bone were located. The calcification volume (CV) and BV/TV could be calculated using the “3D analysis” function in “Custom processing”.

### Alizarin Red staining

For Alizarin Red staining of clinical meniscus tissue sections, the clinical meniscal tissues were routinely dehydrated and embedded in paraffin (clinical meniscus samples were not decalcified). Tissue Section (4 μm) were then prepared according to standard procedures, and staining was performed after deparaffinization. The sections were immersed in Alizarin Red S staining solution (Solarbio, G3280) for 5 min, thoroughly washed with distilled water, and counterstained with hematoxylin to stain the nuclei. After routine dehydration and clearing, neutral resin was used to seal the sections. The stained sections were then observed and photographed under a microscope.

For Alizarin Red staining of calcified nodules after osteoblast induction, the Alizarin Red S Staining Kit for Osteogenesis (Beyotime, C0148S) was used. After the completion of primary meniscal cell differentiation, the cells were fixed for 20 min and washed three times with PBS. Subsequently, Alizarin Red S staining solution was added to evenly cover the cells, and the cells were stained at room temperature for 30 min. After thorough washing with distilled water, the stained cells were observed and photographed under a microscope and camera. For Alizarin Red quantification, 10% cetylpyridinium chloride solution was added to each well, and the samples were shaken slowly on a shaker for 30 min. After dissolving Alizarin Red, the absorbance was measured at 570 nm to quantify calcification.

### Alkaline phosphatase staining

All alkaline phosphatase staining was performed using the Pluripotent Stem Cell BCIP/NBT Alkaline Phosphatase Color Development Kit (Beyotime, C3250S).

For alkaline phosphatase staining of mouse knee joint tissue sections, mouse knee joints were routinely decalcified (Roles-Bio, RBG1110), dehydrated and embedded in paraffin. Tissue section (4 μm) were then prepared according to standard procedures, and staining was performed after deparaffinization. The BCIP/NBT staining working solution was prepared (buffer + BCIP solution + NBT solution) according to the manufacturer’s instructions and added to the section location. The sections were incubated at room temperature for 30–120 min until the desired staining intensity was achieved. The duration of staining was determined at room temperature. The same batch of stained sections was observed under a microscope regularly to confirm the end point of staining. After counterstaining the nuclei with Nuclear Fast Red, the stained sections were observed and photographed under a microscope.

For alkaline phosphatase staining after osteoblast induction, after primary meniscal differentiation was induced, the cells were fixed for 15 min using 4% paraformaldehyde and then washed three times with PBS. Subsequently, the BCIP/NBT staining working solution (as described above) was added, and the cells were stained at room temperature for 30–120 min until the desired staining intensity was achieved. The duration of staining was determined at room temperature as described above. All cells in the same batch were stained for the same duration. After washing with distilled water, the stained cells were observed and photographed under a microscope and camera.

### Immunofluorescence (IF)

For the immunofluorescence staining of clinical meniscus samples, the sections were prepared as described in the ‘Alizarin Red Staining’ section. Antigen repair was performed using citric acid repair solution (P0083, Beyotime) at 65 °C for 16 h, after which the sections were blocked in 10% goat serum for 30 min. Antigen recognition was achieved by incubating primary antibodies against AGEs (bs-1158R, Bioss, RRID: AB_10857744) and RAGE (16346-1-AP, Proteintech, RRID: AB_2878246) overnight at 4 °C and then with goat anti-rabbit Alexa Fluor 488 (Abmart) as a secondary antibody for 30 min at 37 °C. Nuclei were counterstained with DAPI (Beyotime). Different staining methods were used for adjacent sections. According to our μCT results, abnormal calcification in the mouse meniscus still developed from the area of natural mineralization in the anterior horn of the meniscus (Fig. 2c–f). Therefore, we directly selected the anterior horn for observation to accurately locate the calcification area. During the imaging, the AGE staining was uniformly converted from green to red (green → red), and the RAGE staining remained the original color (green) to facilitate the merging of adjacent sections.

For the immunofluorescence staining of mouse knee joint samples, the sections were prepared as described in the ‘Alkaline phosphatase staining’ section. Antigen repair was performed using citric acid repair solution (P0083, Beyotime) at 65 °C for 16 h, after which the sections were blocked in 10% goat serum for 30 min. Antigen recognition was achieved by incubating primary antibodies against AGEs (bs-1158R, Bioss, RRID: AB_10857744), p-mTOR (phospho-mTOR-S2448, 67778-1-IG, Proteintech, RRID: AB_2889842) or ATF4 (60035-1-Ig, Proteintech, RRID: AB_2058598) overnight at 4 °C and then with goat anti-rabbit Alexa Fluor 488 or goat anti-mouse Alexa Fluor 594 (Abmart) secondary antibodies for 30 min at 37 °C. Nuclei were counterstained with DAPI (Beyotime). Different staining methods were used for adjacent sections, and during imaging, ATF4 staining was uniformly converted from red to green (red→green), p-mTOR staining was the same as the original color (red), and AGE staining was uniformly converted from green to cyan (green → cyan) to facilitate the merging of adjacent sections.

### Gene set enrichment analysis (GSEA)

Gene Set Enrichment Analysis was performed using GSEA software (Broad Institute, version 4·3·2). The GSE185064 dataset contains RNA-seq data from healthy and osteoarthritic menisci, and the GSE191157 dataset contains lncRNA and mRNA expression profile information indicating age-related changes in the meniscus. To merge the GSE185064 and GSE191157 datasets, we first used the R package in silicoMerging. The removeBatchEffect function of the R package limma (version 3·42·2) was used to remove batch effects and obtain the final matrix for GSEA.

### Western blotting (WB) analysis

Radioimmunoprecipitation assay (RIPA) lysis buffer was used to extract total protein from cultured cells and meniscus tissues for WB analysis. The proteins were separated via sodium dodecyl sulfate‒polyacrylamide gel electrophoresis (SDS‒PAGE) and wet transferred to polyvinylidene difluoride (PVDF) membranes. The membranes with proteins were subsequently blocked in 5% BSA and then incubated with antibodies specific to AGEs (bs-1158R, Bioss, RRID: AB_10857744), RAGE (16346-1-AP, Proteintech, RRID: AB_2878246), PI3K (60225-1-Ig, Proteintech, RRID: AB_11042594), AKT (60203-2-Ig, Proteintech, RRID: AB_10912803), p-AKT (Phospho-AKT-T308, 29163-1-AP, Proteintech, RRID:AB_2918241), mTOR (66888-1-Ig, Proteintech, RRID:AB_2882219), p-mTOR (Phospho-mTOR-S2448, 67778-1-Ig, Proteintech, RRID: AB_2889842), p38 (14064-1-AP, Proteintech, RRID:AB_2878007), p-p38 (Phospho-p38-Thr180/Tyr182, 28796-1-AP, Proteintech, RRID: AB_2918205), ERK (11257-1-AP, Proteintech, RRID:AB_2139822), p-ERK (Phospho-Thr202/Tyr204, 28733-1-AP, Proteintech, RRID: AB_2881202), β-catenin (51067-2-AP, Proteintech, RRID: AB_2086128), p-β-catenin (Phospho-Ser675, 28853-1-AP, Proteintech, RRID: AB_2923583), ATF4 (10835-1-AP, Proteintech, RRID: AB_2058600), p-ATF4 (Phospho-ATF4-S245, AP0309, ABclonal, RRID:AB_2770925), ubiquitin (10201-2-AP, Proteintech, RRID: RRID:AB_671515), and RUNX2 (ab23981, Abcam, RRID:AB_777785). Subsequently, the membranes were incubated with a secondary antibody conjugated with HRP. Finally, ECL (Millipore) was used to measure the protein bands semiquantitatively, and the results were normalized to the gray value of GAPDH.

### RNA isolation and qRT‒PCR

RNA was extracted from cells and tissues using an RNeasy Kit (R0027, Beyotime) according to the manufacturer’s instructions. Reverse transcription was completed using a Hifair II 1st Strand cDNA Synthesis SuperMix Kit (11123ES60, Yeasen). qRT‒PCR was performed on a real-time PCR system using SYBR dye (Yeasen). mRNA expression was normalized to that of GAPDH, which was used as the housekeeping gene. The relative expression levels of all the qRT‒PCR products were calculated using the ΔΔCt method^27^. The list of primers used in this study is shown in Supplementary Table 2.

### Generation of concentrated lentiviral vectors

For ATF4 knockdown, three short hairpin RNAs (Sh-RNAs) were designed using Genscript, Inc., an online construct builder (see Supplementary Table 3 for shRNA sequences). The lentiviral vector pLVX was cotransfected with pMD2·G (RRID: Addgene_12259) and psPAX2 (RRID: Addgene_12260) using polyethylenimine in HEK293T cells overnight. The culture media was removed and replaced with serum-free media for 3 days, after which the conditioned media was collected and centrifuged to remove cell debris. The viral medium was filtered through a syringe filter (0.45 μM) and purified by ultracentrifugation. Finally, the virus was concentrated, the titer was determined, and the sample was stored at −80 °C in 10 μl aliquots in cold phosphate-buffered saline until use.

### Immunoprecipitation (IP)

Immunoprecipitation was completed using a Universal IP/Co-IP Toolkit (Magnetic Beads) kit (KTD104-CN, Abbkine). Briefly, cells were lysed in nondenaturing lysis buffer supplemented with protease inhibitors and ubiquitin-aldehydes. Cell lysates were diluted in IP buffer, and the anti-ATF4 antibody (10835-1-AP; Proteintech) was covalently bound to Protein A/G magnetic beads. Precipitates were obtained by magnetic racking after overnight incubation and eluted according to the manufacturer’s instructions for blotting analysis.

### High-performance liquid chromatography‒mass spectrometry (HPLC‒MS/MS)

The levels of metabolites were analyzed via LC‒MS methods, as previously described. Cell lysates of differentiated meniscal cells were collected, a one-tenth volume of TCEP working solution (200 mM) was added, and cells were left at room temperature for 15 min. An equal volume of NEM working solution (50 mM) and 1 ml of methanol solution containing the internal standard were added, after which the mixture was allowed to react at −20 °C overnight. After centrifugation at 15,000 rpm for 10 min at 4 °C, 1 ml of the supernatant was concentrated and dried in a vacuum centrifuge.

For HPLC‒MS analysis, the residue was redissolved in 100 μl of methanol/water (1:10, v/v) as the solvent. The concentration of nonessential amino acids was determined using an HPLC (LC-20ADXR, Shimadzu) coupled to a Sciex 3200 MD LC‒MS/MS system (AB Sciex Pte. Ltd.). Chromatographic separation was performed on a Waters X Bridge™ BEH C18 analytical column (2.5 μm, 3.0 × 100 mm; Waters, Torrance, CA) with the mobile phase containing A = 0.1% formic acid in water and B = 0.1% formic acid in methanol by the following program: 0–0.01 min (1% B); 0.01–3 min (1–2% B); 3–6 min (2–90% B); 6–8 min (90–5% B); and 8–10 min (5% B). The injection volume was 10 μl, and the flow rate was 0.4 ml/min. The electrospray ionization (ESI) source was configured with the following conditions for both positive and negative ion modes: Ion Source Gas1 (Gas1) 40, Ion Source Gas2 (Gas2) 60, curtain gas (CUR) 35, source temperature 500 °C, and IonSpray Voltage (ISV) ± 4500 V. The mass spectrometry characteristics of the metabolites are shown in Supplementary Table 4. All HPLC‒MS/MS data were obtained using Analyst Software (v1·6·2).

### Intracellular arginine concentration assay

Intracellular metabolites were obtained by freeze‒thawing samples in liquid nitrogen in a 37 °C water bath. Arginine levels were determined using the ELISA kit (ELK7925, ELK Biotechnology) according to the manufacturer’s instructions. The microplate was coated with purified Arg to make a solid-phase carrier. The sample, standard substance or biotin-labeled antibody was added to the microwell, and then, streptomycin HRP was added after the reaction. Finally, the substrate TMB was used for color development. The absorbance (OD) was measured at a wavelength of 450 nm by a microplate reader, and the sample concentration was calculated.

### Statistical analysis

All the results are expressed as the mean ± SD and were analyzed using GraphPad Prism 9 software. All experiments were repeated at least three times. Student’s *t* test was used to determine the significance of differences between two groups. One-way analysis of variance, followed by Tukey’s test, was used for multiple comparisons. Differences were considered significant at *P < 0.05, **P < 0.01 and ***P < 0.001.

## Results

### AGEs are positively correlated with meniscal calcification

AGEs accumulate in the plasma and tissues of diabetic patients^28^. To assess the role of AGEs in calcification, 20 meniscus samples harvested during total knee arthroplasty procedures were divided into two groups according to the corresponding HbA1c level.

Quantification of the total tissue volume (TV) and calcification volume (CV) of the meniscus was performed using micro-CT, which revealed significantly increased meniscal calcifications in patients with high HbA1c levels (Fig. 1a, b). Furthermore, Alizarin Red and immunofluorescence staining demonstrated that AGEs and RAGE were enriched around the calcification area (Fig. 1c). Next, total RNA and total protein were extracted from the collected tissues. We found higher levels of AGEs and RAGE in the high HbA1c subgroup (Supplementary Fig. 1a–c). We subsequently confirmed the osteogenic ability of human primary meniscal cells through Alizarin Red and ALP staining (Supplementary Fig. 1d, e). WB demonstrated no significant changes in AGE or RAGE protein levels during osteogenic differentiation, indicating that no endogenous AGEs were produced in primary meniscal cells (Supplementary Fig. 1f, g). Based on these findings, we investigated whether AGE-RAGE promotes calcification formation and the underlying mechanisms involved.

### Activation of AGE-RAGE signaling promotes osteogenic differentiation and the mineralization of meniscal cells

To further define the role of AGE-RAGE in the meniscus, we treated human primary meniscal cells with additional AGEs or TTP488 (a RAGE inhibitor) during osteogenic induction and found that AGE treatment significantly increased osteogenic differentiation capacity, while TTP488 treatment decreased osteogenic differentiation capacity (Fig. 2a and Supplementary Fig. 2-1a). Unlike in the previous literature^29^, AGE treatment did not affect RAGE protein levels in meniscal cells (Supplementary Fig. 2-1b, 2-1c), which suggested that the primary impact of AGE-RAGE signaling on meniscal cells was attributed to the presence of AGEs.

To assess the effect of AGE-RAGE in vivo, C57BL/6 J mice were used to establish a destabilized OA model and were administered via an intra-articular knee injection. All mice were randomly divided into the following 4 groups: sham + PBS, sham + AGEs, ACLT + PBS, and ACLT + AGEs. Knee joints were harvested 8 weeks after surgery. Micro-CT analysis revealed that the calcification area in the meniscal region expanded significantly after ACLT and further in the ACLT + AGE group. Similar results were observed in the sham + AGE group (Fig. 2c, d). As shown in Supplementary Fig. 2-2a, AGE injection increased subchondral bone remodeling caused by ACLT. Compared with ACLT, AGE injection further reduced the BV/TV of subchondral bone, suggesting exacerbation of OA progression (Supplementary Fig. 2-2b). Furthermore, safranin O/fast green staining revealed erosion and decreased cellularity of the articular cartilage in all ACLT groups, with the most significant damage observed in the ACLT + AGE injection group (Supplementary Fig. 2-2c). When the Osteoarthritis Research Society International (OARSI) scoring system was used to evaluate osteoarthritis development, similar results were obtained, with the highest scores in the ACLT + AGE injection group (Supplementary Fig. 2-2d).

To evaluate the impact of AGE-RAGE in vivo, db/db mice were utilized as a natural model with high levels of AGEs. All mice were subjected to ACLT surgery. Micro-CT analysis demonstrated that calcification in the meniscal region was more significant in db/db mice than in control mice and was reduced by TTP488 injection. Notably, injection of TTP488 in db/m mice also led to a similar reduction (Fig. 2e, f). The representative micro-CT images in Supplementary Fig. 2-2e demonstrated that TTP488 injection reduced subchondral bone remodeling. TTP488 injection increased the BV/TV of subchondral bone in db/m and db/db mice, suggesting alleviation of OA progression (Supplementary Fig. 2-2f). Similarly, damage to superficial articular cartilage was assessed using safranin O/fast green staining, with the db/db + PBS group exhibiting the most severe damage, while injection of TTP488 alleviated the damage to the cartilage (Supplementary Fig. 2-2g). Consistently, OARSI scoring revealed the highest scores in the db/db + PBS group (Supplementary Fig. 2-2h).

The above results demonstrated that activation of AGE-RAGE signaling enhanced the osteogenic ability of meniscal cells, leading to meniscal calcification and ultimately exacerbating KOA.

### The PI3K-AKT-mTOR signaling pathway is activated by AGE treatment and induces calcification

**Fig. 3:**
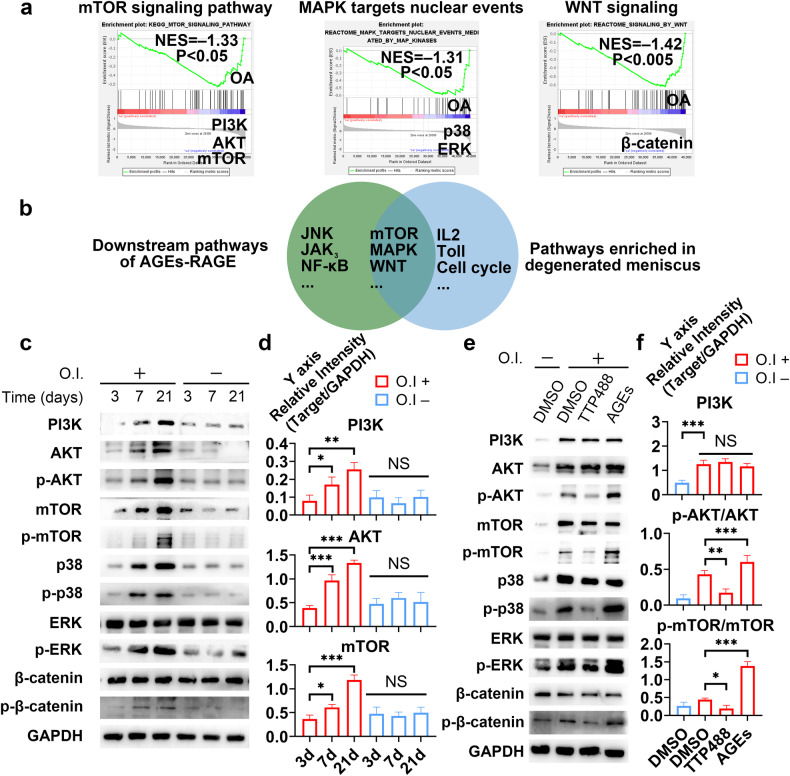
The PI3K-AKT-mTOR signaling pathway is activated by AGE treatment and induces calcification. **a** Enrichment plots from GSEA showing pathways enriched in the degenerated meniscus. **b** Venn diagram showing pathways downstream of AGE-RAGE and pathways enriched in the degenerated meniscus. **c** At different times during osteogenic induction, human primary meniscal cell lysates were analyzed via WB for the indicated proteins. **d** Quantification of the WB data; *n* = 3. **e** Human primary meniscal cells were treated with the indicated drugs during osteogenic induction; cell lysates were analyzed via WB for the indicated proteins. **f** Quantification of the WB data; *n* = 3. O.I. osteogenic induction, which means 21 days of induction when the induction time is not indicated. The data are expressed as the mean ± SD; *P < 0.05; **P < 0.01; and ***P < 0.001. One-way ANOVA was used for comparisons.

We next sought to probe the downstream mechanisms of AGE-RAGE. RAGE activates numerous downstream intracellular signaling pathways, including the mitogen-activated protein kinase (ERK 1/2, p38, and SAPK/JNK), PI3K/AKT/mTOR, JAK/STAT, and Wnt/β-catenin pathways^16,30^. Some of these pathways play pivotal roles in osteogenesis. To further narrow the range of key pathways associated with degenerated meniscal tissue, we conducted gene set enrichment analysis (GSEA) using two GEO datasets (GSE185064 and GSE191157) (Supplementary Fig. 3-1a). The results showed significant enrichment of mTOR signaling, MAPK signaling, WNT signaling, cell cycle checkpoints, IL-2 signaling, and Toll signaling in the degenerated meniscus samples (Fig. 3a and Supplementary Fig. 3-1b). We focused on the first three pathways, given that they have been shown to be influenced by AGE-RAGE signaling (Fig. 3b). We further investigated which pathway might be the most valuable downstream target of AGE-RAGE and responsible for osteogenic differentiation in meniscal cells. We compared the expression of representative genes in different pathways (combined with GSEA results; PI3K/AKT/mTOR for mTOR signaling, p38/ERK for MAPK signaling, and β-catenin for WNT signaling) at different time points during osteogenic induction via WB and qRT‒PCR using human primary meniscal cells. PI3K/AKT/mTOR expression significantly increased at the protein (total protein and phosphorylated protein) and mRNA levels over time (Fig. 3c, d and Supplementary Fig. 3-1c). Concurrently, AGE treatment enhanced the activation of the PI3K/AKT/mTOR pathway, while TTP488 treatment had the opposite effect (Fig. 3e, f). Notably, AGE-RAGE did not significantly affect the total protein or mRNA levels of proteins in the PI3K/AKT/mTOR axis (Fig. 3e, f and Supplementary Fig. 3-1d). We noted the most significant changes in the PI3K/AKT/mTOR axis during osteogenic induction via AGE-RAGE signaling (Fig. 3c–f). We hypothesized that the PI3K/AKT/mTOR pathway plays the most critical role in AGE-RAGE signaling. We subsequently evaluated the effects of a PI3K inhibitor (copanlisib), an AKT inhibitor (MK2206) and an mTOR inhibitor (rapamycin) on the osteogenic differentiation of meniscal cells. Consequently, all the inhibitors markedly inhibited osteogenesis, with the mTOR inhibitor rapamycin demonstrating the most pronounced effect (Supplementary Fig. 3-2). Taken together, these findings indicated that AGE-RAGE signaling enhances the osteogenic differentiation ability of the meniscus via the PI3K/AKT/mTOR axis. To simplify the study design, we substituted the activity of mTOR for the activity of the PI3K/AKT/mTOR axis in subsequent studies.

### ATF4 plays a crucial role in the osteogenic differentiation of meniscal cells and is regulated by AGEs

**Fig. 4:**
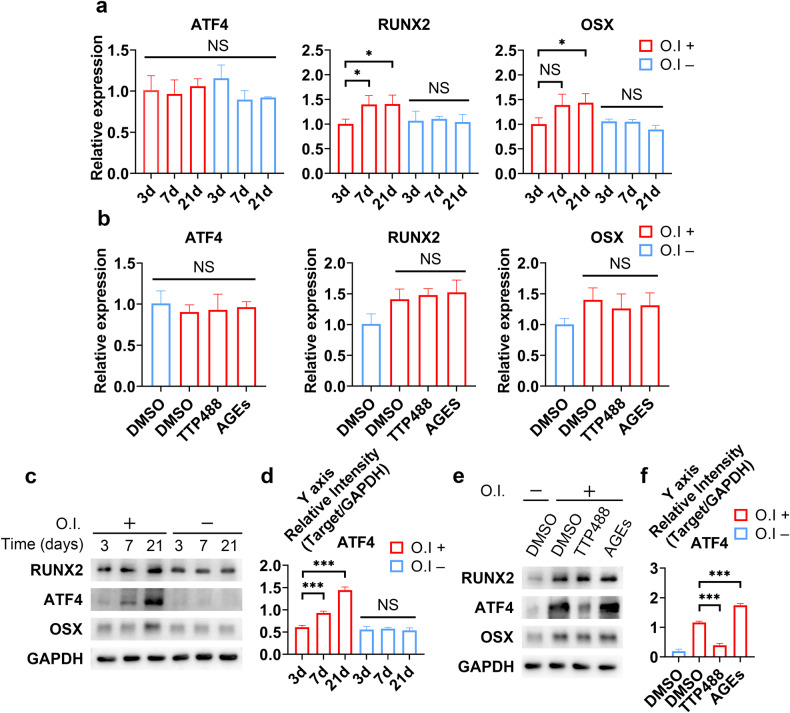
ATF4 plays a crucial role in the osteogenic differentiation of meniscal cells and is regulated by AGEs. **a** After different durations of osteogenic induction, human primary meniscal cell lysates were analyzed via qRT‒PCR for the indicated mRNAs; *n* = 3. **b** Human primary meniscal cells were treated with the indicated drugs during osteogenic induction; cell lysates were analyzed via qRT‒PCR for the indicated mRNAs; *n* = 3. **c** At different times during osteogenic induction, primary meniscal cell lysates were analyzed via WB for the indicated proteins. **d** Quantification of the WB data; *n* = 3. **e** Human primary meniscal cells were treated with the indicated drugs during osteogenic induction; cell lysates were analyzed via WB for the indicated proteins. **f** Quantification of the WB data; *n* = 3. O.I., osteogenic induction, which means 21 days of induction when the induction time is not indicated. The data are expressed as the mean ± SD; *P < 0.05; **P < 0.01; and ***P < 0.001. One-way ANOVA was used for comparisons.

The ability to construct functional bone during the development and maintenance of bone homeostasis relies on the spatiotemporal activation of osteogenic transcription factors. We next sought to explore the function of these osteogenic transcription factors in meniscal cells during osteogenic differentiation that were regulated by AGE-RAGE. By evaluating the changes in the expression of several key components (RUNX2, ATF4, and OSX) during osteogenic induction in human primary meniscal cells treated with or without AGE/TTP488, we found that the mRNA levels of RUNX2 and OSX increased over time and were not affected by AGE-RAGE (Fig. 4a, b). However, the mRNA expression of ATF4 remained stable throughout the process (Fig. 4a, b). Notably, qRT‒PCR analysis revealed that the Ct value for ATF4 was close to that for GAPDH, indicating that the absolute expression level of ATF4 was relatively high. Since it has been shown that ATF4 induces osteogenesis in non-osteoblastic cells and that ATF4 is predominantly subjected to posttranslational regulation^31^, we further evaluated the protein levels of ATF4. WB analysis indicated that the ATF4 protein level increased significantly with osteogenic induction (Fig. 4c, d). Moreover, AGE treatment further upregulated ATF4 protein expression but did not affect the protein levels of RUNX2 or OSX (Fig. 4e, f). These results substantiated the unique role of ATF4 downstream of AGE-RAGE. We next knocked down ATF4 (ATF4 KD) via lentivirus (LV) transfection. After confirming the knockdown efficiency of the construct (Supplementary Fig. 4a–c), we confirmed our hypothesis that ATF4 knockdown significantly inhibited osteogenesis (Supplementary Fig. 4d, e). Hence, we concluded that ATF4 acts as a key transcription factor during the osteogenic differentiation of meniscal cells triggered by AGE stimulation.

### mTOR and ATF4 generate a positive feedback loop to promote osteogenesis in meniscal cells

**Fig. 5:**
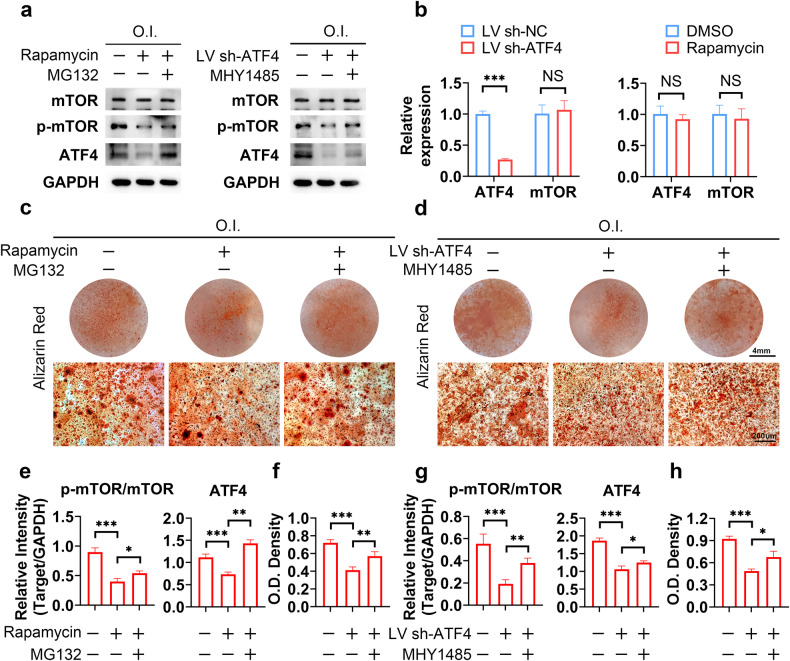
A positive feedback loop involving mTOR and ATF4 promotes osteogenesis in meniscal cells. **a** Human primary meniscal cells were treated with the indicated drugs or with the ATF4 KD lentivirus during osteogenic induction; cell lysates were analyzed via WB for the indicated proteins. **b** Human primary meniscal cells were treated with rapamycin or an ATF4 KD lentivirus (LV) during osteogenic induction; cell lysates were analyzed via qRT‒PCR for the indicated mRNAs; *n* = 3. **c**, **d** Human primary meniscal cells were treated with the indicated drugs or with the ATF4 KD lentivirus (LV) during osteogenic induction; Alizarin Red staining was used to detect osteogenic differentiation. **e**, **g** Quantification of the WB data in (**a**); *n* = 3. **f**, **h** Quantification of Alizarin Red staining; *n* = 3. O.I., osteogenic induction (21 days). The data are expressed as the mean ± SD; *P < 0.05; **P < 0.01; and ***P < 0.001. Student’s *t* test (**b**) and one-way ANOVA (**e**–**h**) were used for comparisons between two groups and multiple groups.

Subsequently, we sought to investigate the interactions between mTOR and ATF4. We found that ATF4 and mTOR had reciprocal effects on each other; mTOR inhibition decreased the total protein level of ATF4, and ATF4 KD led to decreased mTOR activity in human primary meniscal cells (Fig. 5a, e, g). Thus, we speculated that mTOR and ATF4 formed a positive feedback loop during osteogenesis. Considering that no significant difference in mRNA levels was observed (Fig. 5b), the interaction between mTOR and ATF4 may occur at the protein level. During rescue experiments, MG132 was used to induce the overexpression of ATF4, as previously described. Our results showed that MG132 treatment led to ATF4 accumulation in meniscal cells, and ATF4 KD completely reversed the effect of MG132 (Supplementary Fig. 5a–d); these results suggest that although MG132 is not a specific ATF4 agonist, it still promotes osteogenesis by activating ATF4.

Rescue experiments were performed to further confirm our hypothesis. MG132 reversed the effect of rapamycin. Correspondingly, MHY1485, an mTOR agonist, reversed the effect of ATF4 KD (Fig. 5a, e, g). The observed changes in osteogenic capacity were consistent with the changes in protein levels (Fig. 5c, d, f, h). These experiments further demonstrated that mTOR and ATF4 formed a positive feedback loop rather than a pure upstream-downstream relationship.

Interestingly, MG132 treatment alone could not enhance mTOR activity, and MHY1485 treatment alone did not lead to ATF4 accumulation (Supplementary Fig. 5c, d, g, h), suggesting that a regulatory mechanism was switched on to avoid uncontrolled activation of this positive feedback loop. Interestingly, the accumulation of ATF4 induced by MG132 led to an increase in osteogenic capacity, whereas increases in mTOR activation induced by MHY1485 did not achieve the same effect. (Supplementary Fig. 5a, b, e, f). Considering that rapamycin still reduced osteogenic capacity after the induction of excessive ATF4 accumulation with MG132 (Fig. 5a, c–f), we hypothesized that ATF4 plays a crucial role in the positive feedback loop that promotes osteogenesis and that an adequate level of mTOR sustains ATF4 activity.

### mTOR inhibits ATF4 degradation via the ubiquitin‒proteasome pathway

**Fig. 6:**
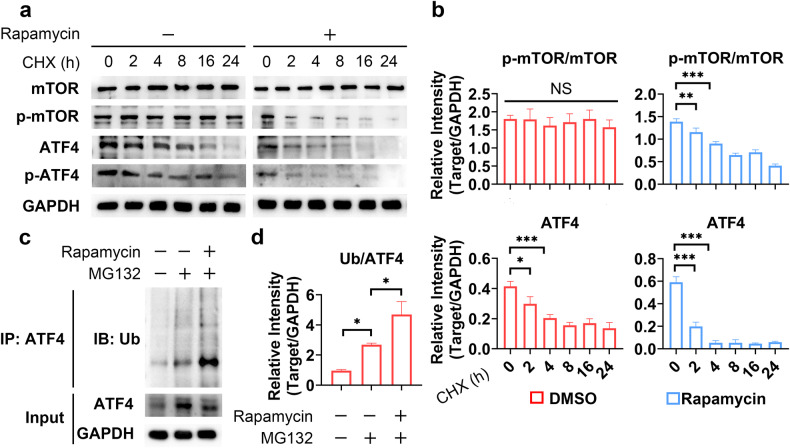
mTOR inhibits ATF4 degradation via the ubiquitin‒proteasome pathway. **a** At different time points after treatment with CHX, human primary meniscal cell lysates were analyzed via WB for the indicated proteins. **b** Quantification of the WB data; *n* = 3. **c** Human primary meniscal cells were treated with the indicated drugs for 24 h; WB analysis of Ub and ATF4 was performed using protein isolated from the ATF4 immunoprecipitate or total lysate. **d** Quantification of the WB data; *n* = 3. The data are expressed as the mean ± SD; *P < 0.05; **P < 0.01; and ***P < 0.001. One-way ANOVA was used for comparisons.

To further investigate the mechanism underlying the mTOR-ATF4-positive feedback loop, we first explored the role of mTOR in regulating ATF4 in human primary meniscal cells. Given that rapamycin did not affect the mRNA level of ATF4 (Fig. 5b), we speculated that mTOR regulates ATF4 post transcriptionally. The ATF4 protein has two phosphorylation sites, Ser219, which is linked to the identification and breakdown of ATF4 by βTrCP and the proteasome^32^, and Ser245, wherein phosphorylation regulates transcriptional activation^33,34^. When mTOR phosphorylates ATF4 at Ser219, there was ATF4 degradation, which resulted in a negative correlation between mTOR activity and the ATF4 protein level. Given the positive correlation between the kinase properties of mTOR and its activity and between mTOR and ATF4 accumulation, we focused on Ser245 phosphorylation. After cycloheximide was used to block protein synthesis, rapamycin treatment accelerated the degradation of ATF4, which was accompanied by a reduction in Ser245 phosphorylation (Fig. 6a, b). Consistent with the findings of previous studies^35^, rapamycin treatment significantly increased ATF4 ubiquitination (Fig. 6c, d), suggesting that mTOR inhibited ATF4 degradation via the ubiquitin‒proteasome pathway.

### ATF4 induces arginine accumulation to activate mTOR in meniscal cells

**Fig. 7:**
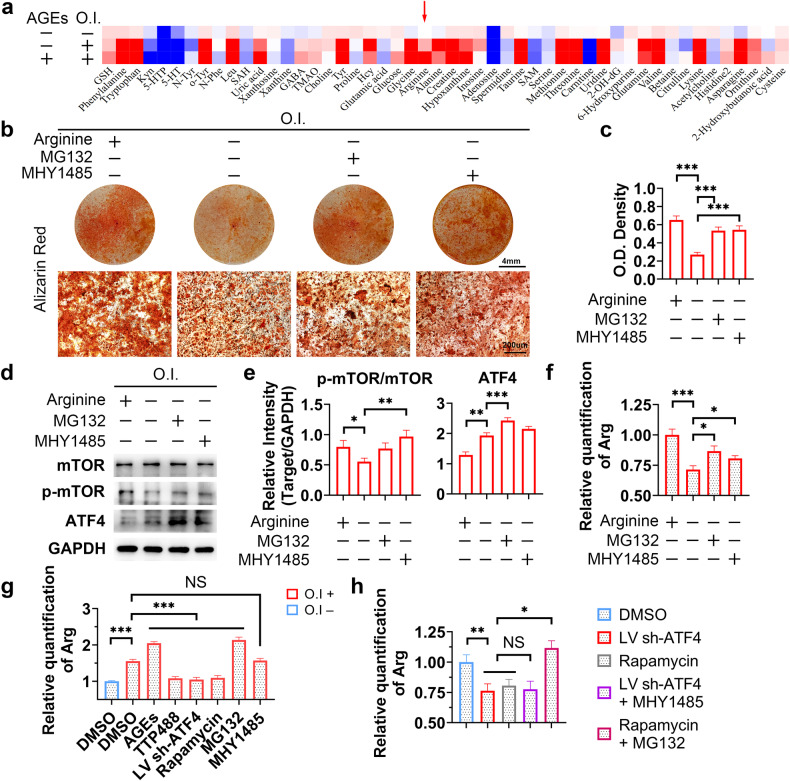
ATF4 induces arginine accumulation to activate mTOR in meniscal cells. **a** A heatmap was generated using mass spectrometry data. The position indicated by the arrow is the arginine, *n* = 3. **b** Human primary meniscal cells were treated with the indicated drugs during osteogenic induction; Alizarin Red staining was used to detect osteogenic differentiation. **c** Quantification of Alizarin Red staining, *n* = 3. **d** Human primary meniscal cells were treated with the indicated drugs during osteogenic induction; cell lysates were analyzed via WB for the indicated proteins. **e** Quantification of the WB data; *n* = 3. **f**–**h** Human primary meniscal cells were treated with the indicated drugs during osteogenic induction; relative quantification of the intracellular arginine concentration was performed via ELISA; *n* = 3. O.I., osteogenic induction (21 days). The data are expressed as the mean ± SD; *P < 0.05; **P < 0.01; and ***P < 0.001. One-way ANOVA was used for comparisons.

We next focused on the mechanism through which ATF4 regulates mTOR activity. ATF4 has been reported to function as a transcription factor^36^. However, no study has reported that ATF4 transcriptionally regulates mTOR. In the present study, we found that ATF4 KD did not affect the overall protein level of mTOR (Fig. 5a). ATF4 is widely recognized as a protein induced by stress that plays a crucial role in promoting the synthesis and absorption of nutrients^37^. Additionally, nutrient availability significantly mediates mTOR^38^. Hence, we hypothesized that amino acid metabolism might be involved in the interaction between ATF4 and mTOR. Multiple amino acids and related metabolites were significantly altered during the osteogenic differentiation of human primary meniscal cells, as was determined by HPLC‒MS/MS (Fig. 7a), and the original results were shown in Supplementary Table 5. However, the levels of only three metabolites, arginine, creatine, and uric acid, were elevated significantly after treatment with AGEs. Arginine caught our interest because the mechanism of mTOR activation by arginine has been well studied^39,40^. The changes in the arginine concentration were further verified by ELISA (Fig. 7g). We found that arginine accumulation could be reversed by TTP488, ATF4 KD, or rapamycin (Fig. 7g). Next, we cultured the cells in arginine-deficient media and discovered that arginine deficiency mitigated the osteogenic differentiation capacity (Fig. 7b, c), which could be reversed by MG132 and MHY1485 treatment (Fig. 7b, c), suggesting that ATF4 or mTOR affects the intracellular metabolism of arginine (Fig. 7f). Interestingly, ATF4 protein expression was upregulated while mTOR activity was inhibited during osteogenic differentiation in arginine-deficient media, indicating that the positive feedback loop was disrupted (Fig. 7d, e). Moreover, excess arginine did not reverse the biological effects of ATF4 KD or rapamycin treatment, as indicated by the inhibition of osteogenic differentiation (Supplementary Fig. 6-1a, 6-1b, 6-2a, 6-2b), decreased ATF4 protein levels and mTOR activity (Supplementary Fig. 6-1c, 6-1d, 6-2c, 6-2d), or decreased intracellular arginine levels (Supplementary Fig. 6-1e, 6-2e). These data suggested that the intracellular arginine concentration was influenced by ATF4 or mTOR rather than by the extracellular arginine concentration.

To further investigate the effect of arginine on the mTOR-ATF4 loop, rescue experiments were performed, and the results demonstrated that MG132 reversed the effect of rapamycin, while MHY1485 did not reverse the effect of ATF4 KD (Fig. 7h); these results demonstrated that arginine accumulation was likely upstream of mTOR and downstream of ATF4 in the regulatory network. Combined with the protein expression pattern in arginine-deficient media (Fig. 7d, e), these findings suggested that arginine accumulation is the result of increased ATF4 protein expression and is also the trigger of mTOR activation. Taken together, these results suggested that ATF4 induces arginine accumulation to activate mTOR.

### AGEs regulate meniscal calcification by activating the mTOR-ATF4-positive feedback loop

**Fig. 8:**
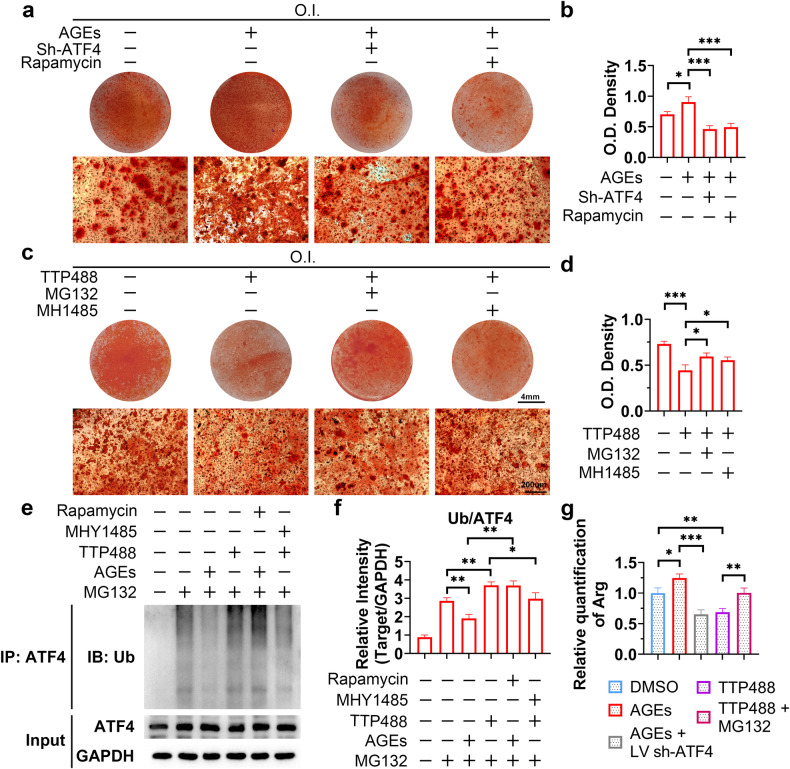
AGEs regulate meniscal calcification by activating the mTOR-ATF4-positive feedback loop. **a**, **c** Human primary meniscal cells were treated with the indicated drugs during osteogenic induction; Alizarin Red staining was used to detect osteogenic differentiation. **b**, **d** Quantification of Alizarin Red staining; *n* = 3. **e** Human primary meniscal cells were treated with the indicated drugs for 24 h; WB analysis of Ub and ATF4 was performed using proteins isolated from the ATF4 immunoprecipitate or total lysate. **f** Quantification of the WB data; *n* = 3. **g** Human primary meniscal cells were treated with the indicated drugs during osteogenic induction, and relative quantification of the intracellular arginine concentration was performed via ELISA; *n* = 3. O.I., osteogenic induction (21 days). The data are expressed as the mean ± SD; *P < 0.05; **P < 0.01; and ***P < 0.001. One-way ANOVA was used for comparisons.

We next sought to verify whether AGE-RAGE induces meniscal calcification by targeting the mTOR-ATF4-positive feedback loop. First, Alizarin Red staining revealed that the increase in osteogenic ability of human primary meniscal cells induced by AGEs could be reversed by ATF4 KD or rapamycin, while the inhibitory effect of TTP488 could be reversed by MG132 or MHY1485 (Fig. 8a–d). WB analyses demonstrated that MG132 and MHY1485 could reverse the decreases in ATF4 protein levels and mTOR activity caused by TTP488, and increases in ATF4 protein levels and mTOR activity caused by AGE treatment could be abolished by ATF4 KD or rapamycin treatment (Supplementary Fig. 7). These results indicated that AGE-RAGE promoted meniscal cell osteogenesis via the mTOR-ATF4-positive feedback loop. In addition, the ubiquitination of ATF4 was regulated by AGEs and blocked by mTOR targeting (Fig. 8e, f). Subsequently, we determined the intracellular arginine concentration and found that the effect of AGE-RAGE on arginine accumulation could also be blocked by targeting ATF4 (Fig. 8g). These results suggested that activation of the ATF4-mTOR positive feedback loop was necessary for the biological effect of AGE-RAGE.

**Fig. 9:**
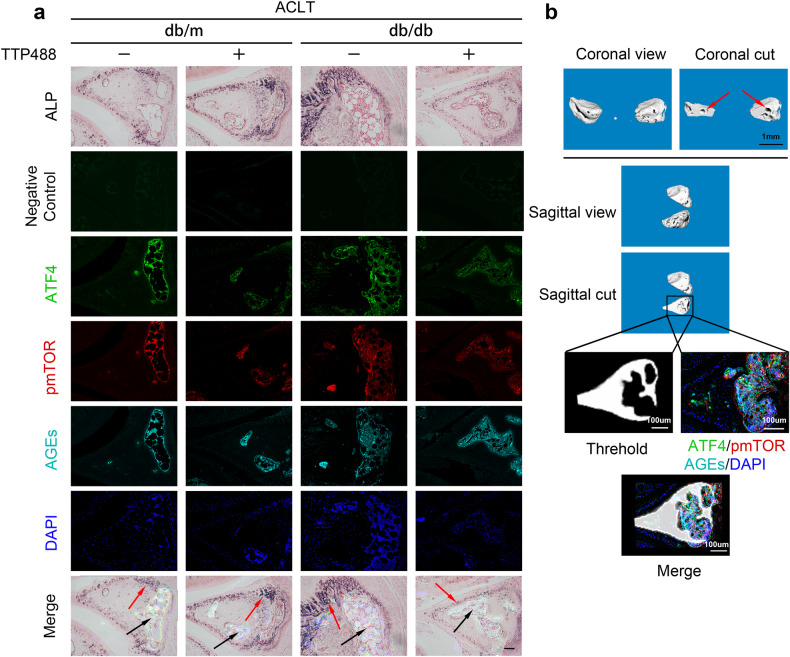
The relationships between the positions of the ATF4-, mTOR-, and AGE-positive areas and calcification areas in the mouse meniscus. **a** Diabetic model and control mice were given different intra-articular knee injections after ACLT surgery. The knee joints were collected at 8 weeks for sectioning. Immunofluorescence analysis and ALP staining of knee joint sections from the different groups. Red arrows, ALP-positive areas. Black arrows, ATF4-, p-mTOR- and AGE-positive areas. Scale bar: 50 μm. **b** Three-dimensional micro-CT images of the meniscal calcification area in the different groups. Red arrows, cavernous structures within the calcification area.

Immunofluorescence microscopy of the meniscal sections consistently revealed that AGEs, p-mTOR, and ATF4 were colocalized (Fig. 9 and Supplementary Fig. 8). ALP staining indicated that ACLT induced the formation of capillary-like structures with cell clusters inside, and calcified areas (grayish-brown flakes) were predominantly found around those capillary-like structures (Fig. 9 and Supplementary Fig. 8). We also observed that intra-articular knee injection of TTP488 decreased the expression of ATF4 and p-mTOR in both the meniscal tissues of db/db and db/m mice (Fig. 9a). The AGE concentration was not significantly affected (Fig. 9a). In contrast, intra-articular knee injection of AGEs significantly increased the AGE concentration, the expression of ATF4, and the p-mTOR level in C57BL/6J mouse meniscal tissue (Supplementary Fig. 8). Interestingly, AGE-, p-mTOR- and ATF4-positive areas appeared to be localized in capillary-like structures rather than overlapping with ALP-positive areas. Three-dimensional reconstructions also revealed many cavernous structures within the calcification area, and ATF4-, p-mTOR- and AGE-positive areas appeared to be localized in these cavernous structures (Fig. 9b). We hypothesize that this phenomenon is related to the proangiogenic effect of AGEs. Neovascularization resulted in the introduction of additional AGEs and cells with elevated levels of the ATF4 protein and mTOR activity, thereby potentially promoting the osteogenesis of meniscal cells. These findings further clarify the close relationship between AGEs and the mTOR-ATF4 loop in vivo.

## Discussion

There is an increasing consensus that meniscal calcification plays a critical role in the initiation and progression of KOA^3,41^. In addition, meniscal calcification is an initial radiological indicator of KOA and can subsequently result in cartilage impairment and precipitate disease progression^8–10^. Therefore, targeting meniscal calcification represents a promising strategy for developing novel therapeutics for KOA. To our knowledge, this study is the first to establish a connection between meniscal cell osteogenesis and the development of meniscal calcification while emphasizing the distinct role of the AGE-RAGE pathway. We also identified a novel positive feedback loop between mTOR and ATF4 during osteogenesis and investigated the underlying mechanism. These findings provide numerous new candidate targets for the treatment of meniscal calcification.

An increasing body of evidence suggests that AGE-RAGE enables bidirectional regulation of osteogenic differentiation. The AGE-RAGE signaling axis promotes vascular calcification but inhibits bone mineralization by targeting multiple pro-osteogenic signaling pathways^42,43^. A similar meniscal bone paradox may exist because meniscal calcification and osteoporosis are commonly observed in elderly individuals^44^. Therefore, we first determined the effect of AGEs on the osteogenic differentiation ability of the meniscus and obtained positive results, which validated our hypothesis. To further analyze the downstream effect of AGE-RAGE, signaling pathways associated with degeneration in meniscal cells were identified by GSEA. After comparing the above pathways with the classical downstream signals of AGEs, we identified the PI3K-AKT-mTOR pathway as the most potent signal transduction pathway.

mTOR is an evolutionarily conserved serine-threonine protein kinase that acts as a critical intracellular molecule to sense extracellular energy status and regulate cell growth and proliferation in various cells and tissues^45^. Several studies have demonstrated that mTOR regulates various biological activities in osteoblasts. In vitro studies have indicated that inhibition of mTOR impairs the proliferation and osteogenic differentiation of BMSCs^46,47^. In vivo also shown that osteoblast-specific deletion of mTOR significantly decreases trabecular bone mass^48^. Likewise, our experiments indicated that inhibition of mTOR led to the suppression of meniscal cell osteogenesis. To investigate the downstream mechanism involved, we further analyzed the expression of key transcription factors involved in the osteogenic differentiation regulatory network. Interestingly, the mRNA level of ATF4 was extremely high in the meniscus. ATF4 is a transcription factor that directly regulates osteogenic differentiation and bone formation^49^. Furthermore, ATF4 can induce osteoblast-specific gene expression in non-osteoblastic cells, including myoblasts, fibroblasts, and even lymphoblasts^31^. Our study further demonstrated that ATF4 affects the osteogenic potential of non-osteoblastic cells, also known as meniscal cells. In contrast with the findings of previous studies, our findings revealed that ATF4 and mTOR form a positive feedback loop rather than a simple upstream-downstream relationship. mTOR and ATF4 have been widely studied for their important roles in regulating oncogenic proliferation and cancer cell survival. The activation of mTOR (especially mTORC1) depends on nutrients and growth factors^50^. GTP-bound RHEB directly binds and activates mTOR in the lysosome in response to nutrient levels, resulting in metabolic reprogramming and cell growth^51^. In contrast, ATF4 is a stress-induced transcription factor that controls the expression of various adaptive genes involved in amino acid transport and metabolism, protection from oxidative stress, and protein homeostasis^52^. ATF4 allows cells to endure periods of stress, such as hypoxia or amino acid limitation^37^. Therefore, ATF4 and mTOR may activate each other theoretically, at least at the functional level. However, ATF4 and mTOR are not activated simultaneously^53,54^. We hypothesize that this is because ATF4 cannot fully alleviate cellular nutrient deficiencies when cells are already under nutrient stress, although ATF4 can promote nutrient uptake. In fact, some studies have revealed that ATF4 induces the expression of mTOR repressors, including SESN2, DDIT4, and REDD1^55^. Our study suggested that ATF4 and mTOR form a positive feedback loop in meniscal cells during osteogenic differentiation. We speculate that this phenomenon is closely related to cellular characteristics and the environment in which the cells are located. Unlike tumor cells, meniscal cells do not exhibit massive proliferation or neovascularization, which are usually observed during meniscal degeneration^56^. We assumed that meniscal cells (especially in the lateral meniscus region) are in a relatively well-nourished environment, which can lead to the formation of a positive feedback loop between ATF4 and mTOR. Consistently, our study revealed that this positive feedback loop is disrupted during culture in arginine-deficient media, as evidenced by increased ATF4 activation and decreased mTOR activation. Accordingly, this topic is worth exploring in the future, not only in this particular field but also in other areas of study.

Moreover, we further investigated how ATF4 and mTOR influence each other. ATF4 is a member of the activating transcription factor (ATF)/cyclic adenosine monophosphate responsive element-binding (CREB) family^36^. While *atf4* mRNA is ubiquitously expressed, the ATF4 protein is unstable and degraded in most cell types through ubiquitination by the βTrCP ubiquitin ligase^35^. The interaction between ATF4 and βTrCP depends on casein kinase-dependent phosphorylation of Ser219 in the βTrCP recognition motif of ATF4^32^, while phosphorylation of Ser245 results in transcriptional activation in osteoblasts and chondrocytes^33,34^. Considering the positive relationship between mTOR activity and the ATF4 protein level, we focused on the phosphorylation of Ser245 and found that mTOR significantly increased phosphorylation. Moreover, the mTOR inhibitor rapamycin markedly increased the ubiquitination of ATF4 and led to its degradation. We next focused on how ATF4 affects mTOR activity. Although ATF4 is widely acknowledged as a transcription factor, our results did not support the supposition that ATF4 regulates the transcription of mTOR. The activation of mTOR is dependent on nutrients and growth factors. Nutrients, especially leucine and arginine, promote the lysosomal localization of mTOR via Ras-related GTP-binding proteins, thereby enabling mTOR to bind to RHEB^38^. Moreover, ATF4 acts at the center of stress signaling, and one of the main functions of ATF4 is to promote the synthesis and uptake of nutrients^37^. On the one hand, ATF4 increases endogenous amino acid availability via autophagy^57^. On the other hand, ATF4 increases exogenous amino acid uptake and increases the expression level of SLC7A1, an essential arginine transporter^58^. Combined with the amino acid metabolomics analysis results, we hypothesized that arginine plays a key role in linking ATF4 and mTOR. As expected, we found that the osteogenic capacity of meniscal cells was significantly reduced during arginine-deficient culture, and this change was accompanied by dysregulation of the ATF4-mTOR positive feedback loop. Notably, ATF4 accumulation contributed to increased intracellular arginine content during arginine-deficient culture, suggesting that ATF4 might also influence endogenous arginine anabolism, such as that which occurs during the urea cycle. In addition, two other metabolites (creatine and uric acid) were further elevated after treatment with AGEs. These genes were also likely involved in the complex regulation of the mTOR-ATF4-positive feedback loop.

The in vivo experiments revealed some interesting results. ACLT and AGE injections induced the formation of capillary-like structures with cell clusters inside the meniscus, while ATF4, mTOR, and AGEs tended to be highly expressed in these cells. Angiogenesis in the meniscus is considered to be one of the features of meniscal injury and can cause knee pain^56^. We hypothesized that meniscal calcification is also closely related to angiogenesis and that neovascularization might be one of the decisive factors in the osteogenic differentiation of meniscal cells. With regard to the reasons for the lack of overlap between ATF4- and p-mTOR-positive regions and calcification areas, we propose the following: (1) cells positive for mTOR-ATF4 promote meniscal cell osteogenesis via paracrine mechanisms; and (2) cells positive for mTOR-ATF4 migrate to the meniscal stroma and undergo osteogenic differentiation, followed by alterations in gene expression. Regrettably, based on the human primary meniscal cells used in our in vitro experiments, we could not distinguish between cells from neovascularization and cells in the meniscal matrix. Given that most of the cells produced during neovascularization are nucleated cells, which differ from conventional blood cells, the origin and characteristics of these cells warrant further investigation.

Several limitations of our study should be acknowledged. First, the AGEs used in this study were produced by reacting BSA with glycolaldehyde under sterile conditions followed by extensive dialysis and purification steps. This is one of the main sources of AGEs^59^. However, clarifying the specific molecules involved is impossible. Moreover, AGEs were directly added to the cell culture medium or injected into the joint in this study. However, the generation of AGEs is complex. The effects of AGEs generated by different routes may be different. The effect of AGEs attached to the matrix may also be different from that of AGEs freely floating in fluid. These questions deserve further study. Second, not all of the human primary meniscal cells used in the in vitro experiments of this study were obtained from the same patient. Although we controlled for some of the demographic data of the patients, it is unlikely that the genetic characteristics of the cells obtained were the same. However, additional studies are needed to validate the results, i.e., establish a reliable meniscal cell line. Moreover, the relationships between ATF4 and mTOR and the role of the positive feedback loop were not further validated in vivo or at the genetic level (e.g., in transgenic animals and in vivo adenoviral infection). In addition, no evidence of a direct interaction between mTOR and ATF4 was found in the present study. Thus, other kinases or even more complex pathways may be involved in the mTOR-mediated regulation of ATF4. Finally, we confirmed that the AGE-RAGE pathway significantly reinforces the mTOR-ATF4-positive feedback loop, but excluding other factors that may limit or enhance this positive feedback loop is important.

**Fig. 10:**
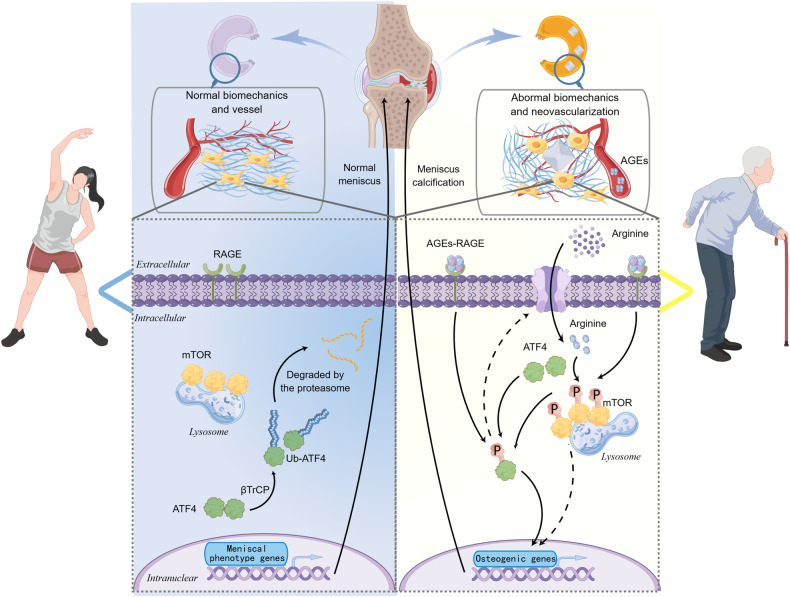
A schematic model of the molecular mechanism underlying the role of AGE-RAGE signaling in the meniscus. In the left panel, a normal meniscus without abnormal neovascularization was observed, exhibiting well-aligned fibers and optimal biomechanical properties. Most of the ATF4 protein in meniscal cells is degraded via the ubiquitination pathway, and there is no significant mTOR activation. The right panel shows that meniscal vessels introduce a significant amount of AGEs with increasing age or glucose levels, which promotes neoangiogenesis and osteogenic differentiation of meniscal cells. AGEs and RAGE activate the ATF4-mTOR positive feedback loop, where mTOR inhibits the degradation of ATF4 by decreasing its ubiquitination. Simultaneously, ATF4 activated mTOR by elevating arginine uptake, eventually leading to calcification of the meniscus and impairment of its biomechanical properties. The schematic was constructed using Figdraw.

In conclusion, we provide undocumented evidence of the facilitative role of AGE signaling in the osteogenic differentiation of meniscal cells and meniscal calcification. Moreover, we found that AGE-RAGE activated an ATF4-mTOR positive feedback loop in which mTOR inhibited ATF4 degradation by reducing its ubiquitination, while ATF4 activated mTOR by increasing arginine uptake (Fig. 10). These data contribute to a better understanding of the pathogenesis of meniscal calcification and provide new insights into treating knee degeneration.

### Supplementary information

Supplementary Tables and Figures

Supplementary Table 5

Unedited blot and gel images

## Data Availability

The datasets used in the GSEA can be accessed in the GEO database by the indicated GSE numbers. The raw mass spectrometry data generated are provided in the supplementary files. All reagents used in this work are available upon request and from a brief statement describing the purpose of their use. The availability of the clinical data generated in the present study is limited because we do not have permission for our informed consent from the research subjects to share data outside our institution without their authorization.
