# Supplementary material for: Advanced glycation end products promote meniscal calcification by activating the mTOR-ATF4 positive feedback loop
Source: Exp Mol Med. 2024 Mar 1;56(3):630–45. doi: 10.1038/s12276-024-01190-6 (PMC10985079; doi:10.1038/s12276-024-01190-6)
Supplement: Supplementary file 1 — Supplementary Tables and Figures [file 12276_2024_1190_MOESM1_ESM.pdf]

Advanced glycation end products promote meniscal calcification by activating the mTOR-ATF4 positive feedback loop

Sheng Yang [1, 2], JiaJun Xie [1], ZhiJie Pan [1, 4], HongMei Guan [5], YueSheng Tu [1], YuanJian Ye [6], ShouBin Huang [6], ShiQiang Fu [7], KangXian Li [1], ZhiWei Huang [8], XiaoQi Li [9], ZhanJun Shi [1], Le Li [3]*, Yang Zhang [1]*

[1]Division of Orthopaedic Surgery, Department of Orthopaedics, Nanfang Hospital, Southern Medical University, Guangzhou, Guangdong, 510515, China.

[2]Department of Orthopedics, 920 Hospital of the Joint Logistic Support Force, Kunming, Yunnan, 650032, China.

[3]Department of Anesthesiology, Zhujiang Hospital, Southern Medical University, Guangzhou, Guangdong, 510282, China.

[4]Department of Orthopaedics, The First People's Hospital of Zhaoqing, Zhaoqing, Guangdong, 526000, China.

[5]Department of Obstetrics and Gynecology, Guangdong Provincial Key Laboratory of Major Obstetric Diseases, The Third Affiliated Hospital of Guangzhou Medical University, Guangzhou, 510150 , China

[6]Department of Orthopaedic, Huizhou First Hospital, Guangdong Medical University, Huizhou, Guangdong, 516003, China

[7]Huizhou First Maternal and Child Health Care Hospital, Huizhou, Guangdong, 516003, China

[8]The First School of Clinical Medicine, Southern Medical University, Guangzhou, Guangdong, 510515, China.

[9]School of Public Health, Southern Medical University, Guangzhou, Guangdong, 510515, China.

*Correspongding authors

Please address all correspondence to:

Yang Zhang, M.D., Ph.D.

Division of Orthopaedic Surgery, Department of Orthopaedics, Nanfang Hospital, Southern Medical University, 1838 Guangzhou Avenue, Guangzhou, Guangdong, 510515, China.

E-mail: nfgjzy@126.com

Le Li, M.D., Ph.D.

Department of Anesthesiology, Zhujiang Hospital, Southern Medical University, Guangzhou, Guangdong, 510282, China.

E-mail: lile11@126.com

Caption for supplementary material

Supplementary Table 1 Stock solution and working solution concentrations for all reagents.

Supplementary Table 2 The sequences of primers.

Supplementary Table 3 The sequences of ATF4 Sh-RNA.

Supplementary Table 4 mTOR and ATF4 form a positive feedback loop to promote osteogenesis in meniscal cells.

Supplementary Table 5 Results of HPLC-MS/MS.

Supplementary Fig.1 AGEs are positively correlated with meniscal calcification.

Supplementary Fig.2-1,2-2 The activation of AGEs-RAGE signaling enhances osteogenic differentiation and mineralization of meniscal cells.

Supplementary Fig.3-1,3-2 PI3K-AKT-mTOR signaling pathway is activated by AGEs treatment and induces calcification.

Supplementary Fig.4 ATF4 plays a crucial role in the osteogenic differentiation of meniscal cells and is regulated by AGEs.

Supplementary Fig.5 mTOR and ATF4 form a positive feedback loop to promote osteogenesis in meniscal cells.

Supplementary Fig.6-1,6-2 ATF4 induces arginine accumulation to activate mTOR in meniscal cells.

Supplementary Fig.7 AGEs regulate meniscal calcification by activating mTOR-ATF4 positive feedback loop.

Supplementary Fig.8 The position relationship between ATF4, mTOR, AGEs positive areas and calcification areas in mouse meniscus.

Supplementary Fig.9 The effects of different reagents (working solution) on the viability of human primary meniscal cells.

Supplementary Tables

Supplementary Table 1 Stock solution and working solution concentrations for all reagents

Name	Stock solution (mM)	Working solution (nM)
TTP488	2.5	1500
AGEs	20mg/ml	50ug/ml
Copanlisib	0.5	50
MK2206	10	200
Rapamycin	5	5
MHY1485	10	400
MG132	5	50

Supplementary Table 2 The sequences of primers

Name	Forward (5'to3')	Reverse (5'to3')
ATF4	ATGACCGAAATGAGCTTCCTG	GCTGGAGAACCCATGAGGT
GAPDH	GGAGCGAGATCCCTCCAAAAT	GGCTGTTGTCATACTTCTCATGG
RUNX2	CCGCCTCAGTGATTTAGGGC	GGGTCTGTAATCTGACTCTGTCC
AKT	GTCATCGAACGCACCTT·CCAT	AGCTTCAGGTACTCAAACTCGT
mTOR	TCCGAGAGATGAGTCAAGAGG	CACCTTCCACTCCTATGAGGC
PI3KR	ACCACTACCGGAATGAATCTCT	GGGATGTGCGGGTATATTCTTC
OSX	CCTCTGCGGGACTCAACAAC	AGCCCATTAGTGCTTGTAAAGG
p38	CTGTTGGACGTTTTTACACCTGC	AGACCTCGGAGAATTTGGTAGA
β-catenin	CATCTACACAGTTTGATGCTGCT	GCAGTTTTGTCAGTTCAGGGA

Supplementary Table 3 The sequences of ATF4 Sh-RNAs

Name	Sequence (5'to3')
Sh ATF4 # 1	GGGTATAGATGACCTGGAAACTTCAAGAGAGTTTCCAGGTCATCTATACC CTTTTTT
Sh ATF4 # 2	GGTGAACCCAATTGGCCATCTTTCAAGAGAAGATGGCCAATTGGGTTCAC CTTTTTT
Sh ATF4 # 3	GGATATCACTGAAGGAGATAGTTCAAGAGACTATCTCCTTCAGTGATATC CTTTTTT

Supplementary Table 4 The mass spectrometry characteristics of metabolites

	Q1 Mass (Da)	Q3 Mass (Da)	DP (Volts)	EP (Volts)	CE (Volts)	CXP (Volts)
Dopamine	154.2	137.2	28.33	10.46	15.49	3.35
GSH	433.1	304.1	51.85	4.98	21.77	4.47
Phenylalanine	166.1	120.1	39.26	3.74	19.12	2.53
Tryptophan	205.2	188.1	38.16	3.12	15.78	3.25
Kyn	209.1	192.1	37.55	3.04	15.89	3.25
5-HTP	221.1	204.3	41.47	3.11	15.86	3.52
5-HT	177.1	160.1	29.85	4.18	14.05	3.87
N-Tyr	227.1	181	34.69	5.28	17.37	3.02
o-Tyr	182.1	136.1	40	10	20	3
N-Phe	194.2	91.1	46.68	4.41	31.2	2.88
Leu	132.1	86.1	37.08	3.25	11.78	2.87
Proline	116.1	116.1	20	10	5	5
SAH	385.1	136.2	42.89	3.74	30.58	4.77
Uric acid	169.1	169.1	20	10	5	3
Xanthosine	285	153	40	10	20	3
Xanthine	153	110	59.65	8.15	23.84	2.47
GABA	104.2	87.2	32	4.49	12.36	3
TMAO	76.13	58.3	51	7.07	22.97	2.43
Choline	104.45	60.2	46.96	6.43	21.55	2.46
Tyr	182.1	136.2	32.4	4.22	16.94	2.46
Proline	116.19	70.2	34.73	3.97	20.02	2.71
Hcy	136.1	118	21.46	6	12.4	3
Glutamic acid	148.18	84.1	33.87	3.09	20.47	2.48
Glucose	203.1	203.1	49.67	4.75	5	15.87
Glycine	76	58.2	43.35	3.94	23.83	3
Arginine	175.2	70.2	41.28	4.22	31.1	2.58

Alanine	90.1	72.1	32.17	4.2	8.2	2.78
Creatine	132	90	40	10	15	3
Hypoxanthine	137	94	40	10	15	3
Inosine	269.1	137	208	2.97	19.01	2.52
Adenosine	268.1	118.9	40	10	15	3
Spermidine	146.1	146.1	40	10	5	3
Taurine	126	108.1	47	7.16	16.01	3.81
SAM	399.1	250.1	30.14	4.82	21.16	3.89
Serine	106.1	60.2	33.76	6.04	17.7	2.53
Methionine	150.05	56.3	36.14	3.05	21.52	2.42
Threonine	119.94	73.9	30.38	4.04	12.01	2.76
Carnitine	162.5	103.1	20.37	2.9	20.37	3
Uridine	245.2	113.2	256.51	3.06	15.56	3.6
2-OH-dG	268.1	152.2	33.85	3.48	15.61	3.09
6-Hydroxypurine	137.1	119.2	59.7	11.41	25.8	2.94
Glutamine	147.1	84.1	33.19	2.98	22.61	3.04
Valine	118.1	72.2	37.12	4.54	14.05	2.69
Betaine	118.2	59.1	47.41	4.71	23.19	2.4
Citrulline	176.11	159.1	32.36	4.04	15.11	2.87
Lysine	147.19	84.1	32.29	5.91	20.19	3.19
Acetylcholine	146.1	87.1	39.5	2.98	19.79	2.99
Histidine2	156.3	109.8	40.81	10.74	19.76	2.26
Asparagine	133	74.1	35.91	3.32	18.7	2.88
Ornithine	133.1	70	31.49	3.14	22.84	2.59
2-Hydroxybutanoic acid	105.1	59.2	28.36	8.7	9.23	2.44
Cysteine	247.1	158.1	48.78	5.36	25.67	2.46
IS	171.1	125.22	16.51	10.47	19.14	3

	171.3	125	35.62	5.98	17.98	3.31

Supplementary Table 5 Results of mass spectrometry (It will be provided in Excel format)

Supplementary Figures

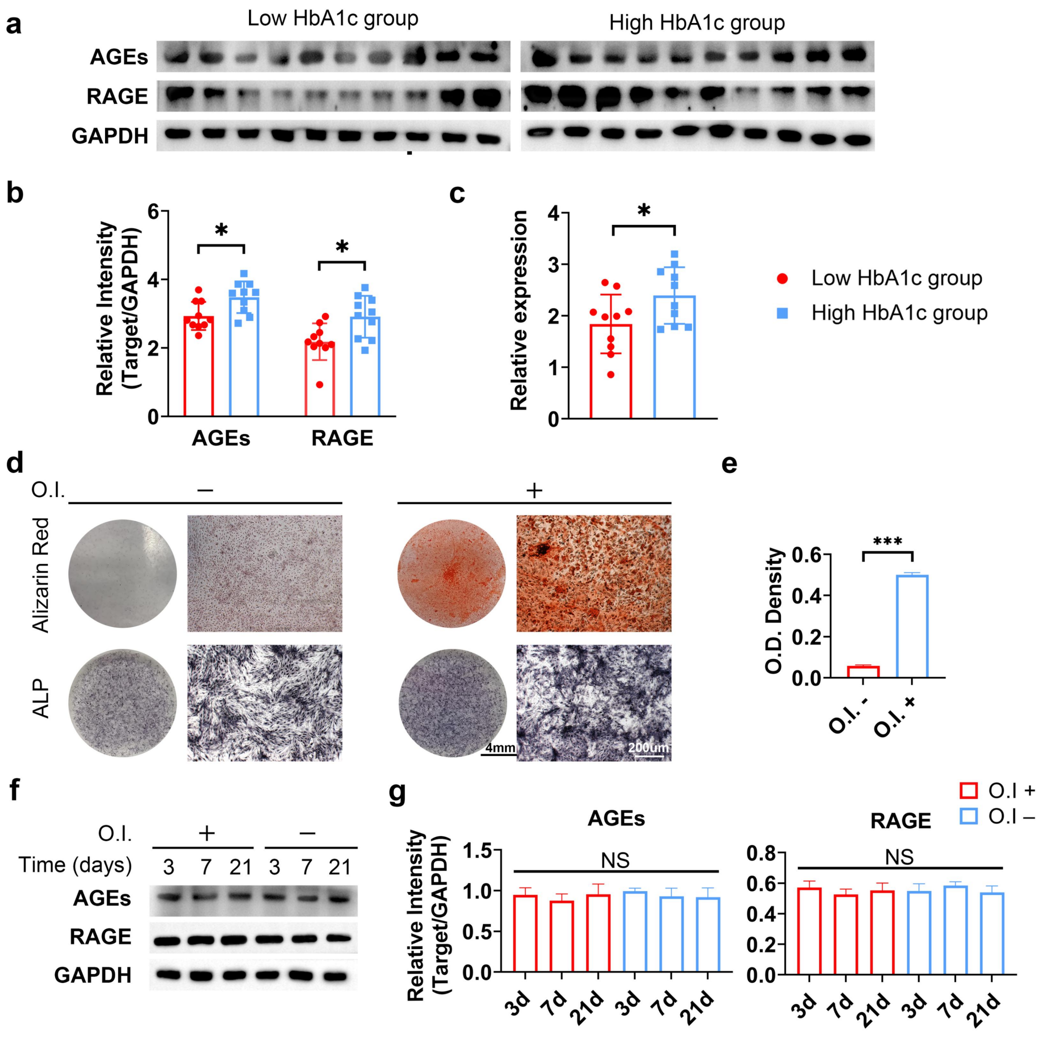

Supplementary Fig. 1 AGEs are positively correlated with meniscal calcification.

(a) WB analysis of AGEs and RAGE in different groups of clinical meniscal samples. (b) Intensity on WB was quantified by densitometric analysis using ImageJ software, n=10; (c) qRT-PCR analysis of RAGE in different groups of clinical meniscal samples. n=10. (d) Osteogenic differentiation detected by Alizarin Red and ALP, showing the osteogenic ability of meniscal cells. (e) Quantification of alizarin red staining, n=3. (f) At different times during osteogenic induction, human primary meniscal cell lysates were analyzed by WB for the indicated proteins; (g) Quantification of WB, n=3. O.I., osteogenic induction, it means 21 days of induction when induction time is not indicated. Data are expressed as mean ± SD, * $P < 0.05$; ** $P < 0.01$; *** $P < 0.001$. Student's t-test and two-way ANOVA were used for comparison between two groups (b, c, e) and multiple groups (g).

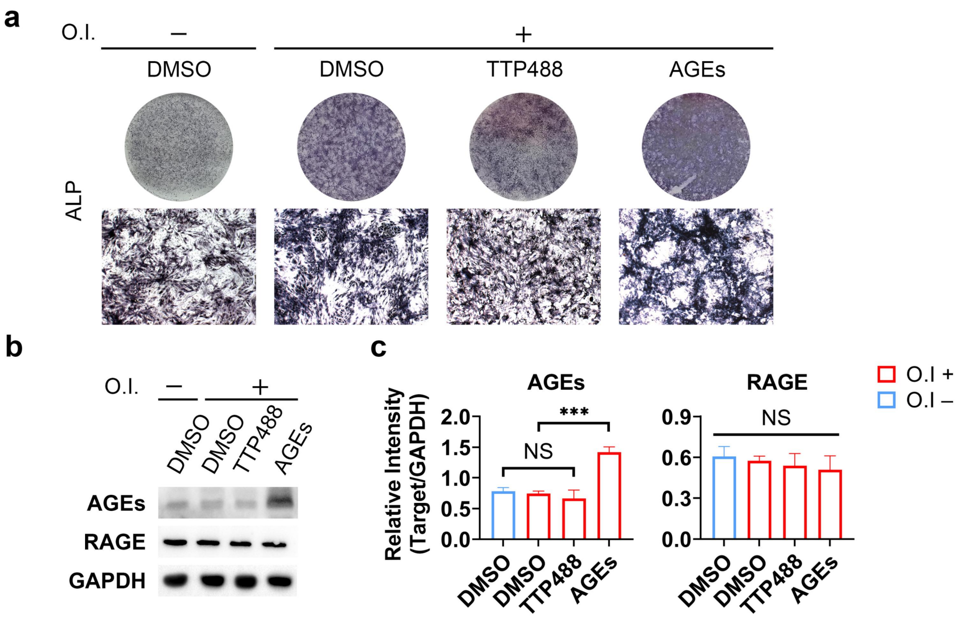

Supplementary Fig. 2-1 The activation of AGEs-RAGE signaling enhances osteogenic differentiation and mineralization of meniscal cells. (a) Human primary meniscal cells were treated with indicated drugs during osteogenic induction, and ALP staining were used to detect osteogenic differentiation. (b) Human primary meniscal cells were treated with indicated drugs during osteogenic induction; cell lysates were analyzed by WB for the indicated proteins. (c) Quantification of WB, n=3. O.I., osteogenic inductin (21 days). Data are expressed as mean ± SD, * P< 0·05; ** P< 0·01; *** P< 0·001. One-way ANOVA was used for comparisons.

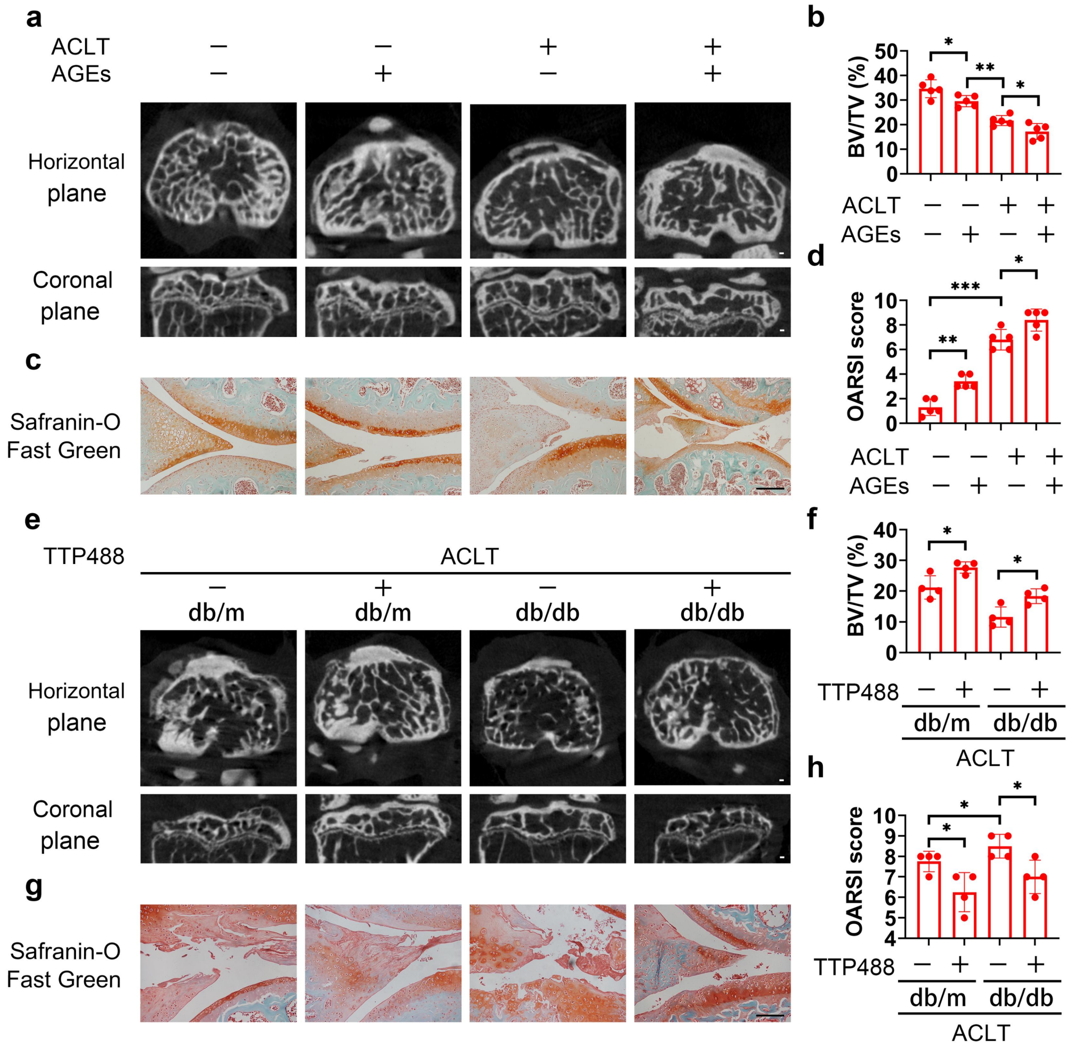

Supplementary Fig. 2-2 The activation of AGEs-RAGE signaling enhances osteogenic differentiation and mineralization of meniscal cells. (a) Coronal and horizontal micro-CT images of proximal tibia in different C57bl/6J mouse groups. Scale bar: 100 µm. (b) Quantification of bone volume fraction (BV/TV) of subchondral bone, n=5. (c) Safranin O/fast green staining of knee joint sections. (d) OARSI scoring assessment of specimen, n=5. Scale bar: 100 µm. (e) Coronal and horizontal micro-CT images of proximal tibia in different diabetic model and control mouse groups. Scale bar: 100 µm. (f) Quantification of bone volume fraction (BV/TV) of subchondral bone, n=4. (g) Safranin O/fast green staining of knee joint sections. Scale bar: 100 µm. (h) OARSI scoring assessment of specimen, n=4. Data are expressed as mean ± SD, * $P< 0.05$; ** $P< 0.01$; *** $P< 0.001$. Two-way ANOVA were used for comparisons.

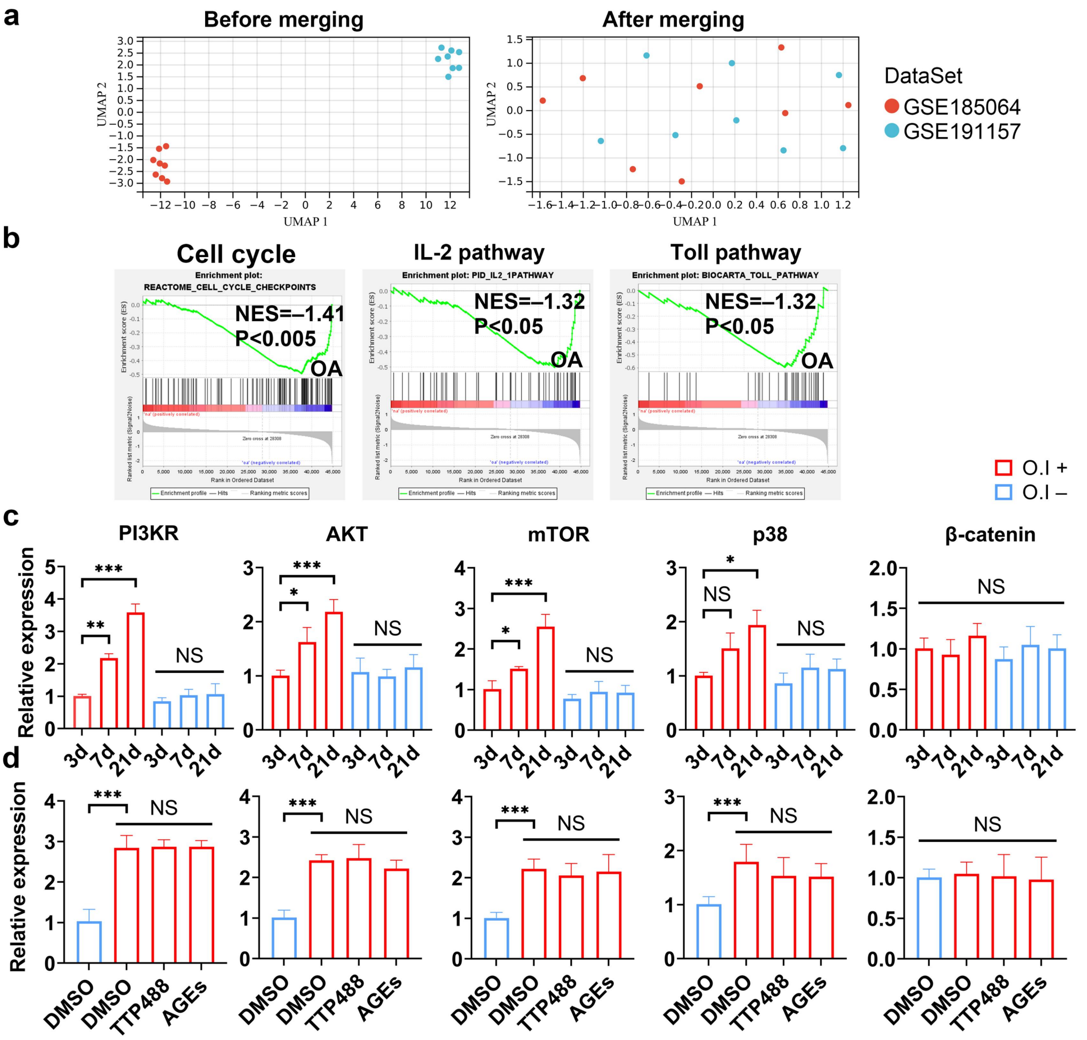

Supplementary Fig. 3-1 PI3K-AKT-mTOR signaling pathway is activated by AGEs treatment and induces calcification. (a) Uniform Manifold Approximation and Projection (UMAP) plot from analysis of datasets using R software inSilicoMerging and limma. (b) Enrichment plots from GSEA show pathways enriched in the degenerated meniscus. (c) At different times during osteogenic induction, human primary meniscal cell lysates were analyzed by qRT-PCR for the indicated mRNA, n=3. (d) Human primary meniscal cells were treated with indicated drugs during osteogenic induction; cell lysates were analyzed by qRT-PCR for the indicated mRNA, n=3. O.I., osteogenic induction (21 days). Data are expressed as mean ± SD, * P< 0·05; ** P< 0·01; *** P< 0·001. One-way ANOVA was used for comparisons.

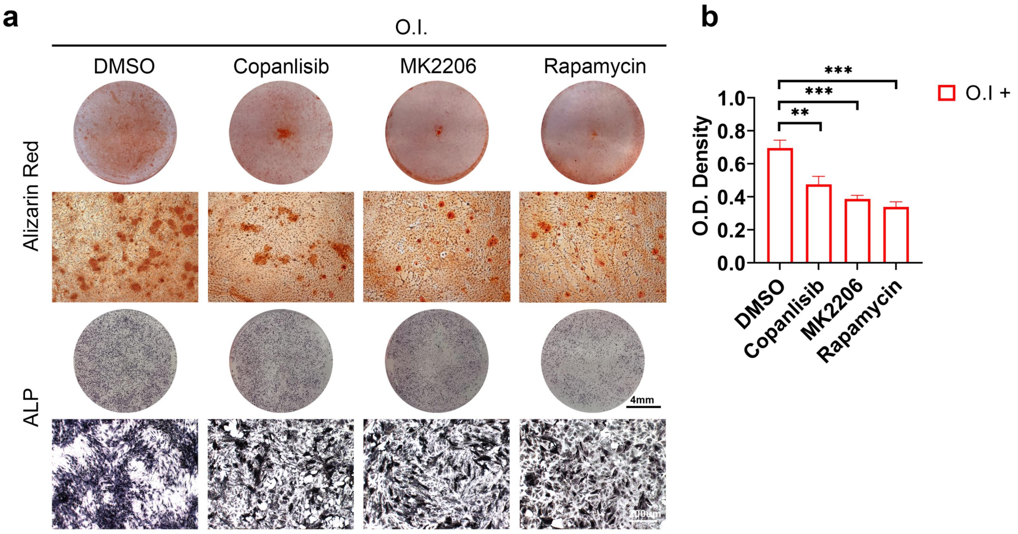

Supplementary Fig. 3-2 PI3K-AKT-mTOR signaling pathway is activated by AGEs treatment and induces calcification. (a) Human primary meniscal cells were treated with indicated drugs during osteogenic induction, and Alizarin Red and ALP staining were used to detect osteogenic differentiation. (b) Quantification of alizarin red staining, n=3. O.I., osteogenic induction (21 days). O.I., osteogenic induction (21 days). Data are expressed as mean ± SD, * P< 0·05; ** P< 0·01; *** P< 0·001. One-way ANOVA was used for comparisons.

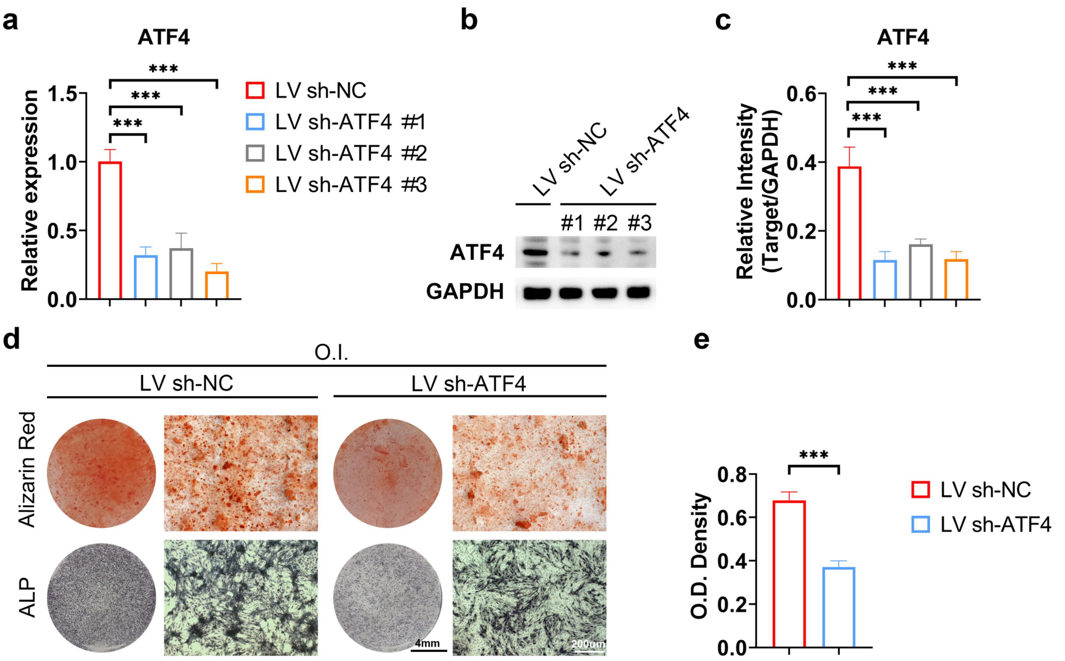

Supplementary Fig. 4 ATF4 plays a crucial role in the osteogenic differentiation of meniscal cells and is regulated by AGEs. (a, b) Human primary meniscal cells were treated with LVs; cell lysates were analyzed by WB and qRT-PCR to evaluate the efficiency of LVs, n=3. (c) Quantification of WB, n=3. (d) Human primary meniscal cells were treated with ATF4 KD LV; Alizarin Red and ALP staining were used to detect osteogenic differentiation. (e) Quantification of Alizarin Red, n=3. O.I., osteogenic induction, it means 21 days of induction when induction time is not indicated. Data are expressed as mean ± SD, * P< 0·05; ** P< 0·01; *** P< 0·001. Student's t-test (e), one-way ANOVA (a, c) were used for comparison between two groups and multiple groups.

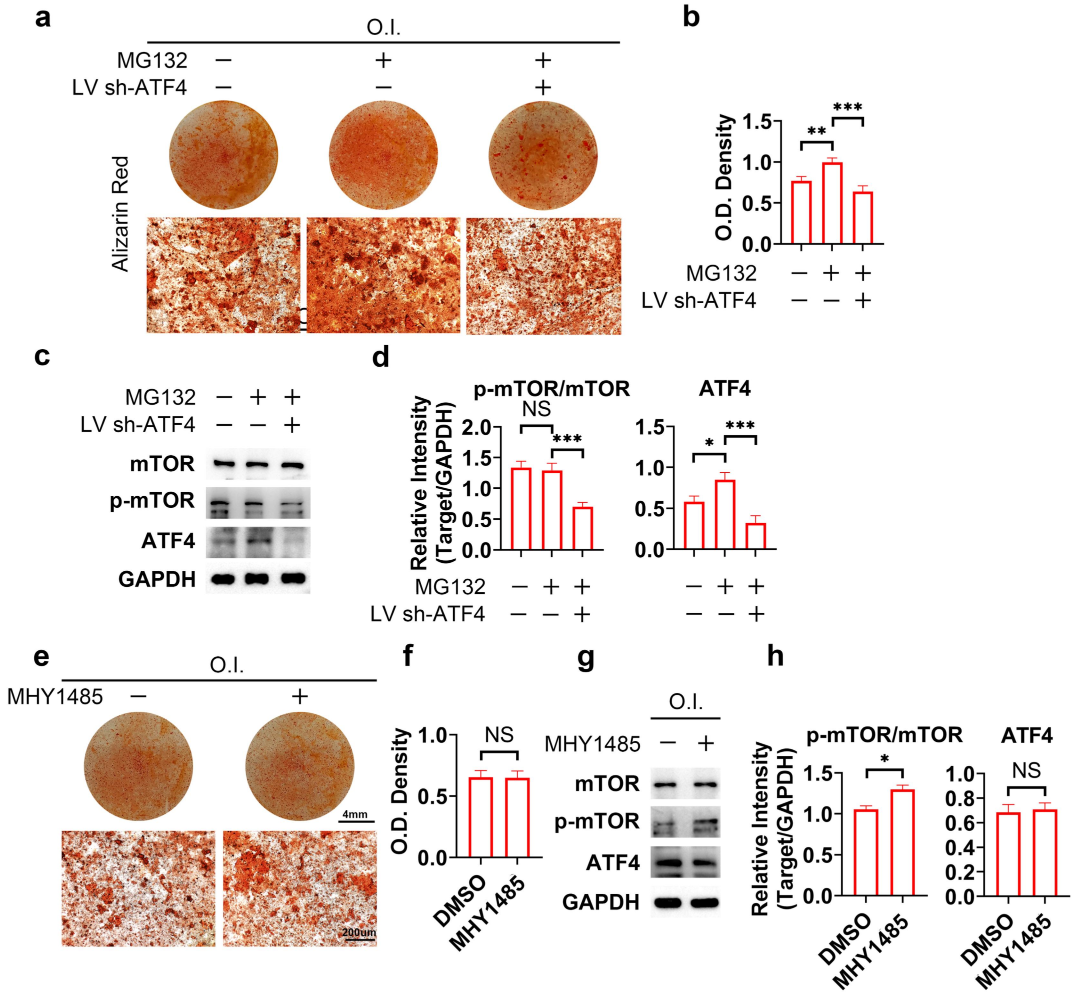

Supplementary Fig. 5 mTOR and ATF4 form a positive feedback loop to promote osteogenesis in meniscal cells. (a, e) Human primary meniscal cells were treated with indicated drugs or ATF4 KD LV during osteogenic induction, and Alizarin Red staining was used to detect osteogenic differentiation. (b, f) Quantification of Alizarin Red staining, n=3. (c, g) Human primary meniscal cells were treated with indicated drugs or ATF4 KD LV during osteogenic induction; cell lysates were analyzed by WB for the indicated proteins. (d, h) Quantification of WB, n=3. All results were expressed as mean ± SD. O.I., osteogenic induction (21 days). Data are expressed as mean ± SD, * P< 0·05; ** P< 0·01; *** P< 0·001. Student's t-test (f, h), one-way ANOVA (b, d) were used for comparison between two groups and multiple groups.

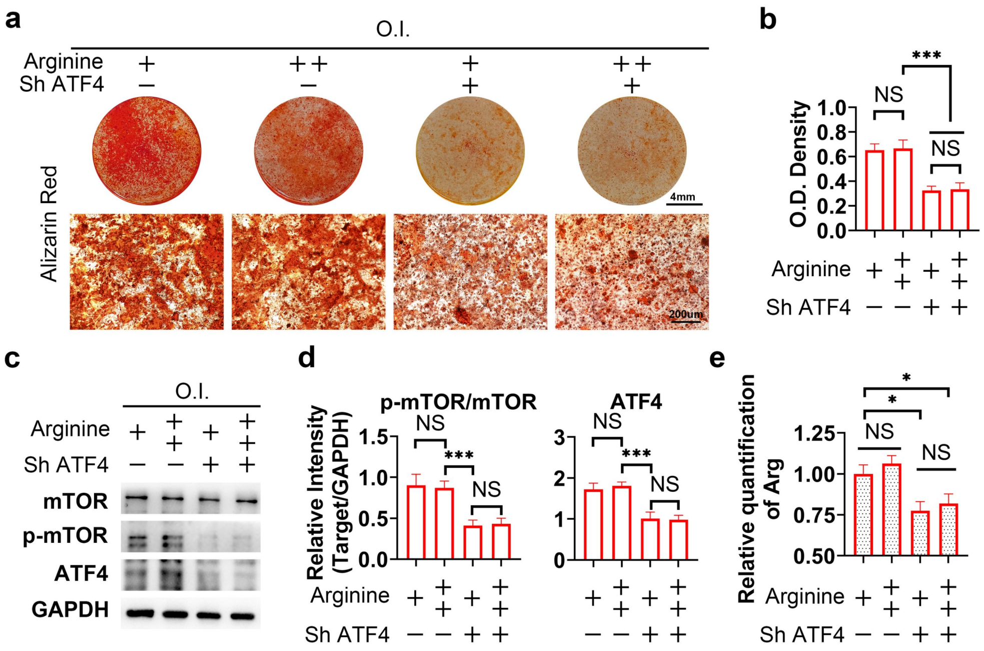

Supplementary Fig. 6-1 ATF4 induces arginine accumulation to activate mTOR in meniscal cells.

(a) Human primary meniscal cells were treated with indicated drugs during osteogenic induction; Alizarin Red staining was used to detect osteogenic differentiation. (b) Quantification of Alizarin Red staining, n=3. (c) Human primary meniscal cells were treated with indicated drugs during osteogenic induction; cell lysates were analyzed by WB for the indicated proteins. (d) Quantification of WB, n=3. (e) Human primary meniscal cells were treated with indicated drugs during osteogenic induction, and relative quantification of intracellular arginine was performed using ELISA, n=3. O.I., osteogenic induction (21 days). Arg++ means that the arginine concentration is twice that of the conventional medium (168 mg/L). Data are expressed as mean ± SD, * P< 0·05; ** P< 0·01; *** P< 0·001. One-way ANOVA was used for comparisons.

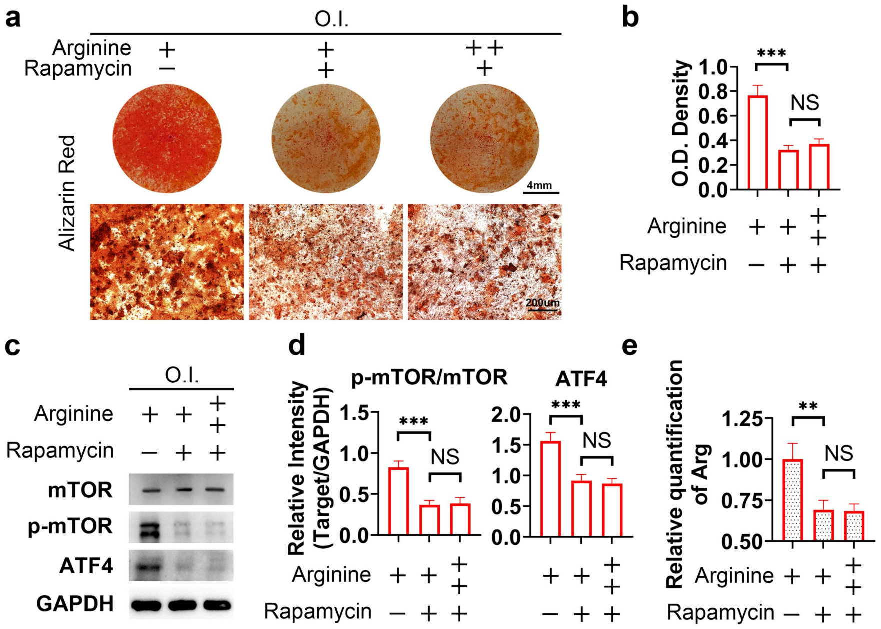

Supplementary Fig. 6-2 ATF4 induces arginine accumulation to activate mTOR in meniscal cells.

(a) Human primary meniscal cells were treated with indicated drugs during osteogenic induction; Alizarin Red staining was used to detect osteogenic differentiation. (b) Quantification of Alizarin Red staining, n=3. (c) Human primary meniscal cells were treated with indicated drugs during osteogenic induction; cell lysates were analyzed by WB for the indicated proteins. (d) Quantification of WB, n=3. (e) Human primary meniscal cells were treated with indicated drugs during osteogenic induction, and relative quantification of intracellular arginine was performed using ELISA, n=3. O.I., osteogenic induction (21 days). Arg++ means that the arginine concentration is twice that of the conventional medium (168 mg/L). Data are expressed as mean ± SD, * P< 0·05; ** P< 0·01; *** P< 0·001. One-way ANOVA was used for comparisons.

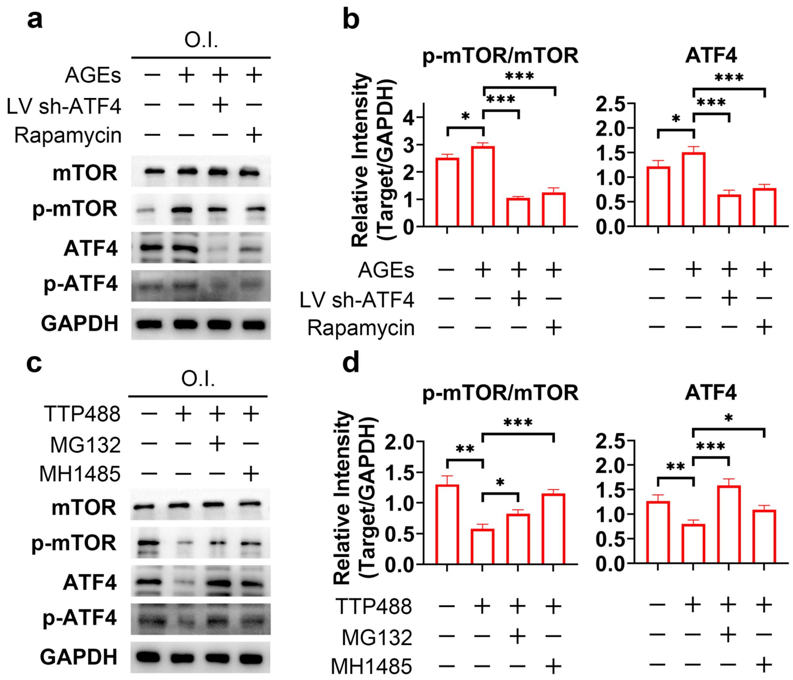

Supplementary Fig. 7 AGEs regulate meniscal calcification by activating the mTOR-ATF4 positive feedback loop. (a, c) Human primary meniscal cells were treated with indicated drugs during osteogenic induction; cell lysates were analyzed by WB for the indicated proteins. (b, d) Quantification of WB, n=3. O.I., osteogenic induction (21 days). Data are expressed as mean ± SD, * $P < 0{\cdot}05$; ** $P < 0{\cdot}01$; *** $P < 0{\cdot}001$. One-way ANOVA was used for comparisons.

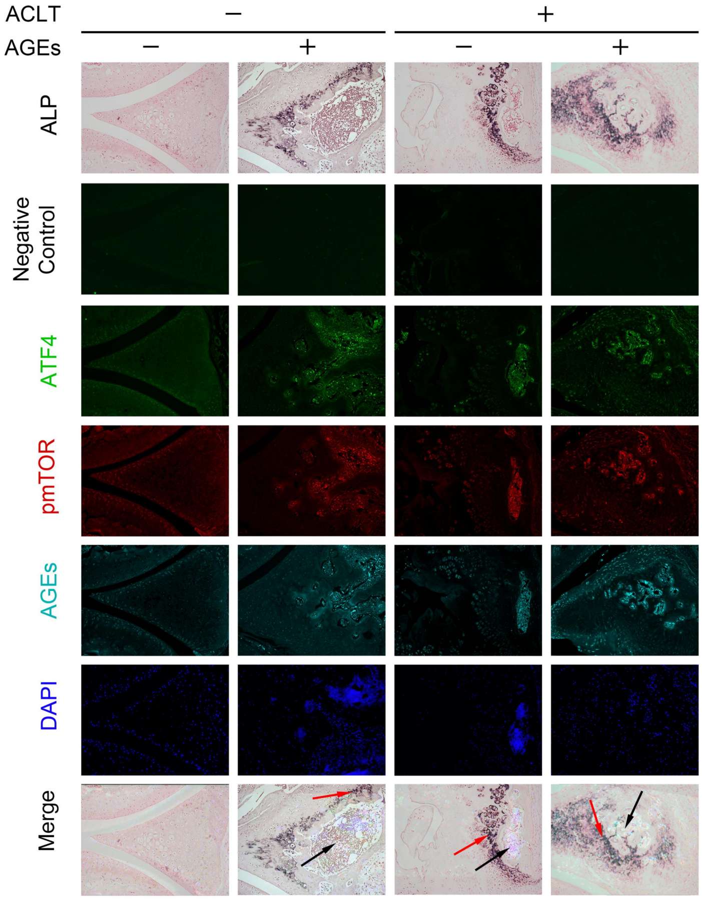

Supplementary Fig. 8 The position relationship between ATF4, mTOR, AGEs positive areas and calcification areas in mouse meniscus. C57BL/6J mice were given different intra-articular knee injections after sham or ACLT surgery. The knee joints were collected at 8 weeks for sectioning. Immunofluorescence analysis and ALP staining of knee joint sections in different groups. Red arrows, ALP-positive areas. Black arrows, ATF4, p-mTOR and AGEs-positive areas. Scale bar: 50 μm.

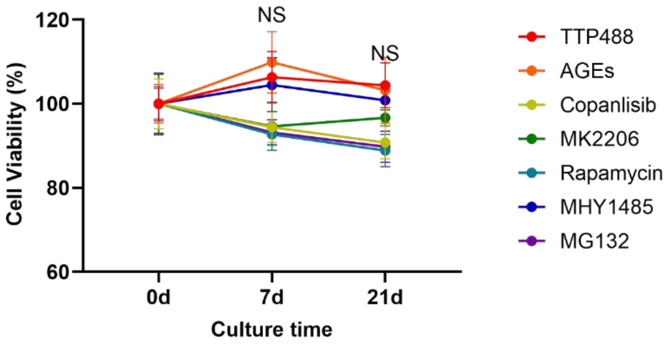

Supplementary Fig. 9 The effects of different reagents (working solution) on the viability of human primary meniscal cells. The human primary meniscal cells were grown to 90% confluence (i.e., ready for osteogenic induction), which was recorded as day 0. Subsequently, different reagents were added and cell activity was measured by CCK8 assay on days 7 and 21. Data are expressed as mean ± SD, One-way ANOVA were used for comparisons.
