# Supplementary material for: Advanced glycation end products promote meniscal calcification by activating the mTOR-ATF4 positive feedback loop
Source: Exp Mol Med. 2024 Mar 1;56(3):630–45. doi: 10.1038/s12276-024-01190-6 (PMC10985079; doi:10.1038/s12276-024-01190-6)
Supplement: Supplementary file 3 — Unedited blot and gel images [file 12276_2024_1190_MOESM3_ESM.pdf]

# Advanced glycation end products promote meniscal calcification by activating the mTOR-ATF4 positive feedback loop

Sheng Yang [1, 3], ZhiJie Pan [1], HongMei Guan [4], YuanJian Ye [5], ShouBin Huang [5], ShiQiang Fu [6], YueSheng Tu [1], KangXian Li [1], ZhiWei Huang [3], XiaoQi Li [7], ZhanJun Shi [1], Le Li [2]*, Yang Zhang [1]*

[1]Division of Orthopaedic Surgery, Department of Orthopaedics, Nanfang Hospital, Southern Medical University, Guangzhou, Guangdong, 510515, China.

[2]Department of Anesthesiology, Zhujiang Hospital, Southern Medical University, Guangzhou, Guangdong, 510282, China.

[3]The First School of Clinical Medicine, Southern Medical University, Guangzhou, Guangdong, 510515, China.

[4]Department of Obstetrics and Gynecology, Guangdong Provincial Key Laboratory of Major Obstetric Diseases, The Third Affiliated Hospital of Guangzhou Medical University, Guangzhou, 510150 , China

[5]Department of Orthopaedic, Huizhou First Hospital, Guangdong Medical University, Huizhou, Guangdong, 516003, China

[6]Huizhou First Maternal and Child Health Care Hospital, Huizhou, Guangdong, 516003, China

[7]School of Public Health, Southern Medical University, Guangzhou, Guangdong, 510515, China.

*Correspongding author

Please address all correspondence to:

Yang Zhang, M.D., Ph.D.

Division of Orthopaedic Surgery, Department of Orthopaedics, Nanfang Hospital, Southern Medical University, 1838 Guangzhou Avenue, Guangzhou, Guangdong, 510515, China.

E-mail: nfgjzy@126.com

Le Li, M.D., Ph.D.

Department of Anesthesiology, Zhujiang Hospital, Southern Medical University, Guangzhou, Guangdong, 510282, China.

E-mail: lile11@126.com

Supplementary Figure 1A

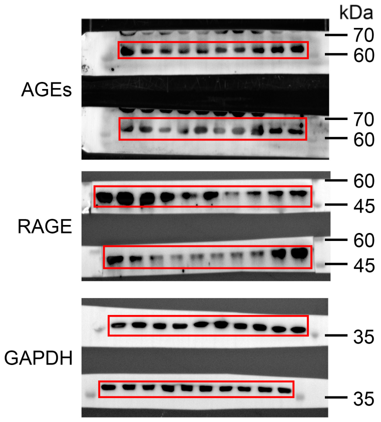


Supplementary Figure 1F

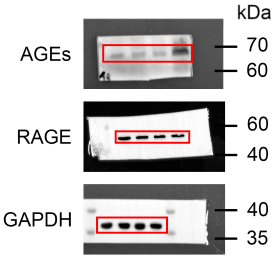


Figure 3C

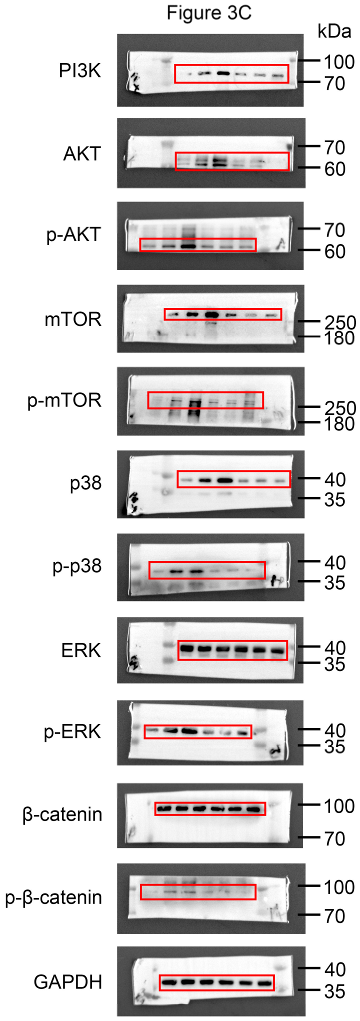


Figure 3E

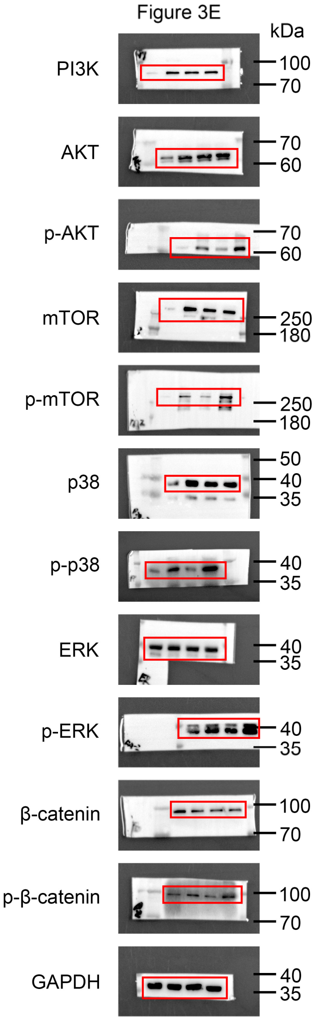

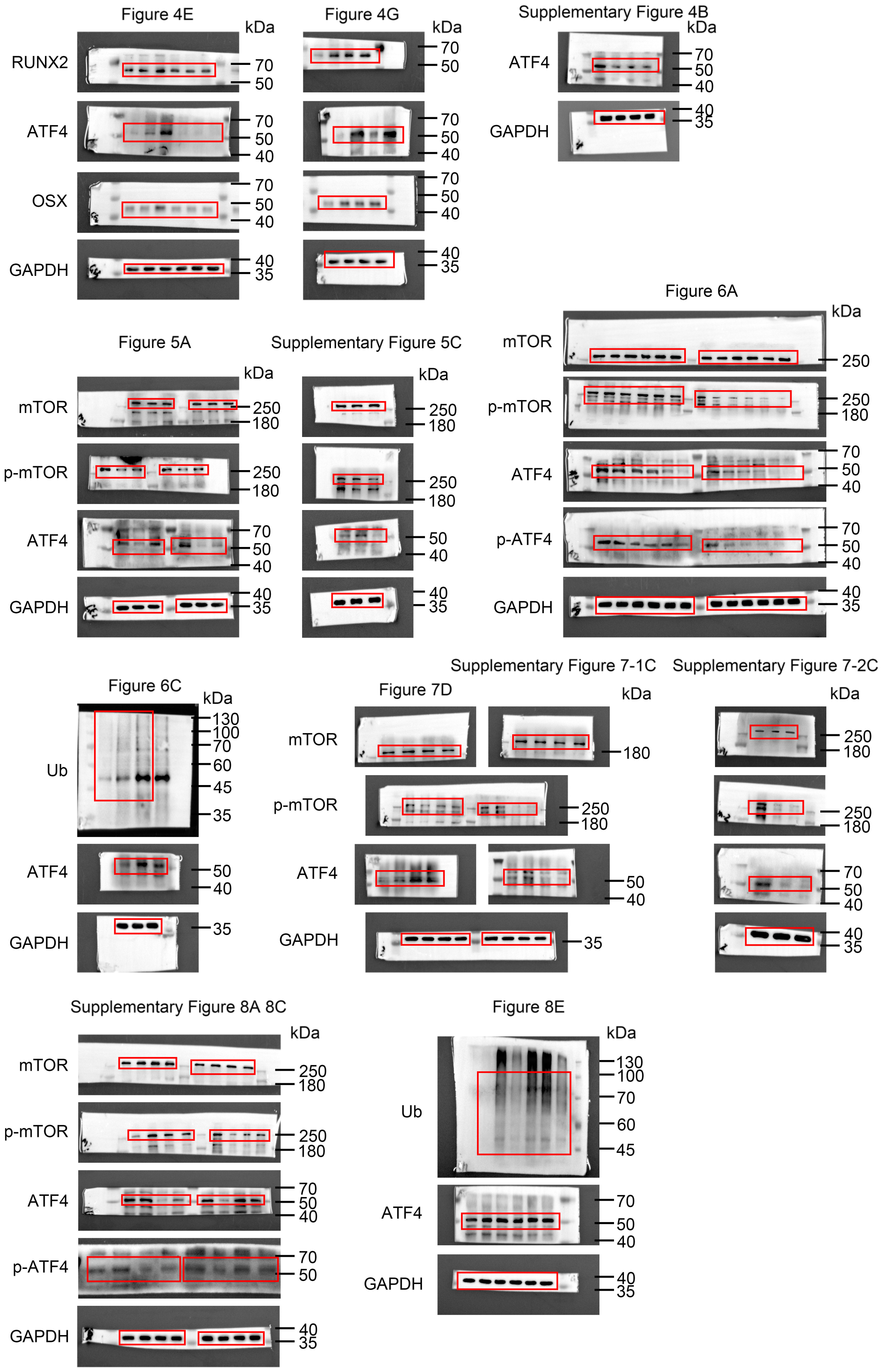


Uncropped blots for western blotting that appeared in the manuscript. All uncropped blot were detected by Tanon 5200 system.
